# Inulin ameliorates chronic ketamine-induced anxiety-like behaviors and impairments in spatial learning and memory: involvement of gut microbiota, microbial metabolite short-chain fatty acids, and the BDNF-TrkB-ERK1/2-CREB pathway

**DOI:** 10.3389/fmicb.2026.1765079

**Published:** 2026-04-07

**Authors:** Zhilong Xu, Jie Zhang, Canrun Hu, Yayan Luo

**Affiliations:** 1 Institute of Neuropsychiatry, The Affiliated Brain Hospital, Guangzhou Medical University, Guangzhou, Guangdong, China; 2 Guangdong Engineering Technology Research Center for Translational Medicine of Mental Disorders, Guangzhou, China; 3 Department of Psychosomatic Medicine, The Affiliated Brain Hospital, Guangzhou Medical University, Guangzhou, Guangdong, China

**Keywords:** BDNF, gut microbiota, inulin, ketamine, SCFAs, schizophrenia

## Abstract

Chronic ketamine exposure results in psychotic and cognitive symptoms that resemble those found in patients with schizophrenia. Emerging evidence suggests that patients with schizophrenia exhibit gut microbiota dysbiosis and decreased levels of short-chain fatty acids (SCFAs) and BDNF, which are related to the severity of psychotic and cognitive symptoms. Dietary inulin can regulate gut microbiota, SCFAs, and BDNF. However, the role of gut microbiota, SCFAs, and BDNF in chronic ketamine-induced schizophrenia-like behaviors is unclear. In this study, we found that chronic ketamine exposure for 28 days caused gut microbiota dysregulation, reduced the expression of SCFAs in serum, hippocampus, and feces, elevated gut permeability, downregulated the BDNF-TrkB-ERK1/2-CREB signaling pathway, caused neuronal damage, and decreased the expression of synaptic proteins Syn and PSD-95, which may lead to anxiety-like behaviors, prepulse inhibition (PPI) deficits, and spatial learning and memory deficits. In addition, inulin intervention reversed gut microbiota dysbiosis by decreasing the abundance of *Colidextribacter*, *Oscillibacter*, *Alistipes*, and *Desulfovibrio*, while increasing the abundance of *Lachnospiraceae_NK4A136_group*, *Faecalibaculum*, and *Blautia*. It also increased the expression of SCFAs, alleviated gut barrier damage, and upregulated the BDNF-TrkB-ERK1/2-CREB signaling pathway to reduce neuronal damage and enhance the expression of Syn and PSD-95, which may improve chronic ketamine-induced anxiety-like behaviors, PPI impairments, and spatial learning and memory deficits. Our findings provide additional evidence that inulin may be a promising candidate for treating chronic ketamine-associated anxiety-like behaviors and deficits in spatial learning and memory, as well as behavioral deficits in schizophrenia with dysbiosis.

## 1 Introduction

Schizophrenia is a common and devastating psychotic disorder characterized by a wide range of clinical symptoms, including positive, negative, and cognitive symptoms, which manifest in late adolescence and early adulthood (Swerdlow et al., 2018). However, the etiology of schizophrenia remains poorly understood. Ketamine is a derivative of phencyclidine (PCP), and its main pharmacological mechanism is as a non-competitive antagonist of N-methyl-D-aspartate (NMDA) receptors. Acute and chronic administration of ketamine recapitulates positive, negative, and cognitive symptoms similar to those seen in schizophrenia in both humans and rodents (Braff et al., 2001; Luo et al., 2021). Therefore, ketamine administration has been used as a pharmacologic model to mimic schizophrenia (Ahmed et al., 2018). However, the molecular mechanism by which chronic ketamine induces schizophrenia-like behaviors is still not fully understood.

Gut microbiota disturbance has been reported to play an important role in the development of schizophrenia (Yuan et al., 2025). The intestinal microbiome can modulate nervous system development, synaptic structure and function, neurotransmitter metabolism, and neuroendocrine functions via the gut–brain axis, which may contribute to the psychopathology of schizophrenia (Guo et al., 2021; Xu et al., 2025a). Previous clinical studies have found gut microbiota dysbiosis in schizophrenia patients, which is associated with cognitive impairments, including visual learning and memory, processing speed, and verbal learning (Zhu et al., 2025a; Zhu et al., 2025b). It has been reported that *Bifidobacterium breve A-1* intervention for 4 weeks alleviates anxiety and depression symptoms in patients with schizophrenia (Okubo et al., 2019). In the Poly I: C mouse model of schizophrenia, prenatal Poly I: C exposure causes aberrant gut microbiota composition in offspring of both sexes during adolescence and adulthood, which may be correlated with anxiety-like behaviors and prepulse inhibition (PPI) deficits following prenatal Poly I: C exposure (Xu et al., 2025b). Moreover, transplantation of gut microbiota derived from patients with schizophrenia induces schizophrenia-like behaviors in mice, including hyperactivity, anxiety-like behaviors, impaired social interaction, and memory deficits (Wei et al., 2024). These findings suggest that gut microbiota plays a crucial role in the psychotic and cognitive symptoms of schizophrenia.

Aberrations in short-chain fatty acids (SCFAs) and brain-derived neurotrophic factor (BDNF) levels are found in patients with schizophrenia, correlated with abnormal gut microbiota composition (Yang et al., 2025; Deng et al., 2022). SCFAs, mainly acetic acid, propionic acid, and butyric acid, are formed through the fermentation of dietary fiber by the intestinal microbiome in the small intestine and colon (Yang et al., 2022; Lu et al., 2022). SCFAs maintain the integrity and permeability of the blood–brain barrier (BBB) and gut mucosal barrier by modulating the expression of tight junction proteins, such as zonula occludens-1 (ZO-1) and Occludin (Maqsood and Stone, 2016). Additionally, SCFAs can cross the BBB and regulate neurotransmitter production, immune system function, and synaptic plasticity, exerting extensive influences on the central nervous system (CNS) (Suda and Matsuda, 2022; D'Amato et al., 2020). A recent clinical study has shown that schizophrenia patients exhibit decreases in fecal levels of acetic acid, propionic acid, butyric acid, isobutyric acid, isovaleric acid, and isohexanoic acid, which are correlated with changes in gut microbiota (Deng et al., 2022). Meanwhile, patients with schizophrenia show reductions in serum levels of caproic acid and valeric acid, and reduced serum caproic acid is positively associated with immediate memory (Peng et al., 2022). Furthermore, gut microbiota plays an important role in the expression of BDNF (Guo et al., 2021; Maqsood and Stone, 2016). BDNF is a crucial neurotrophic factor that primarily affects the proliferation and differentiation of hippocampal neurons, as well as learning and memory (Nieto et al., 2013). Reductions in BDNF levels may contribute to psychotic symptoms and cognitive deficits in schizophrenia through dysfunction of synaptic transmission and plasticity (Nieto et al., 2013; Shi et al., 2022). BDNF binds to tropomyosin receptor kinase B (TrkB), which phosphorylates the extracellular signal-regulated protein kinase 1/2 (ERK1/2) on Thr202/Tyr204. The phosphorylated ERK1/2 then enters the nucleus and phosphorylates cAMP-response element-binding protein (CREB) on Ser133, modulating the expression of downstream target genes associated with synaptic integrity, synaptic plasticity, hippocampal neurogenesis, and cognition (Guo et al., 2015; Du et al., 2023). The disturbance of gut microbiota is associated with abnormal BDNF levels in schizophrenia patients (Yang et al., 2025). Clinical studies have demonstrated that serum and hippocampal BDNF levels are reduced in patients with schizophrenia, and these reductions in serum levels are significantly correlated with positive and negative symptoms, as well as cognitive impairments in attention, perceptual-motor skills, processing speed, and memory (Dwivedi et al., 2003; Carlino et al., 2011). Taken together, these findings suggest that SCFAs and BDNF are potential mediators between gut microbiota and schizophrenia and that gut microbiota may be involved in schizophrenia by modulating SCFA and BDNF expression.

Inulin, a natural polysaccharide found in plants, is used in functional food, medicine, and many other fields as a dietary fiber and prebiotic (Guo et al., 2021). Inulin can be fermented and metabolized by the gut microbiome in the colon into SCFAs, including acetic acid, propionic acid, and butyric acid (Liu et al., 2020). Meanwhile, inulin can restore gut dysbiosis by increasing the abundance of probiotics and decreasing the abundance of pathogenic bacteria, thus exerting beneficial modulatory effects such as improving cognitive deficits, alleviating neuronal necrosis, and preventing synaptic damage (Guo et al., 2021; Xu et al., 2025a; Zou et al., 2024). Several previous studies have shown that inulin attenuates anxiety-like behaviors as well as impairments in PPI, recognition memory, and spatial learning and memory by restoring gut microbiota, elevating SCFA levels, alleviating neuronal necrosis, upregulating the BDNF-TrkB signaling pathway, and enhancing the length of the postsynaptic density (Guo et al., 2021; Xu et al., 2025a; Liu et al., 2020; Morshedi et al., 2020). These findings suggest that inulin treatment is a potential intervention strategy for preventing and treating diseases related to gut microbiota disorders and nervous system dysfunctions. Given the important role of gut microbiota, SCFAs, and BDNF in schizophrenia, inulin may be a prominent candidate for the treatment of schizophrenia.

Previous studies have demonstrated that chronic ketamine users show decreases in serum BDNF levels and schizophrenia-like abnormal behaviors, such as deficits in PPI, emotional indifference, and impairments in spatial problem solving and verbal memory (Cheng et al., 2018; Ke et al., 2014; Braff et al., 2001). Few studies have researched the chronic effects of ketamine on gut microbiota and microbial metabolite SCFAs (Xu et al., 2025a; Xie et al., 2024; Wan et al., 2022). The correlations between gut microbiota and chronic ketamine-induced schizophrenia-like behaviors and BDNF levels remain largely unclear. As far as we know, no study has investigated the potential relationship between gut microbiota, SCFA, and BDNF levels in chronic ketamine-induced schizophrenia-like behaviors. In the present study, we investigated the molecular mechanism underlying chronic ketamine-induced schizophrenia-like behaviors. We aimed to determine whether chronic ketamine exposure caused changes in gut microbiota composition, impaired gut barrier integrity, altered the expression of SCFAs and BDNF, and resulted in schizophrenia-like behavioral deficits, including anxiety-like behaviors, PPI deficits, and impairments in spatial learning and memory. We also explored the signaling pathway associated with these changes. Furthermore, we evaluated whether inulin could attenuate behavioral disorders by regulating gut microbiota, SCFAs, and BDNF.

## 2 Methods and materials

### 2.1 Animals

Adult male C57BL/6 mice aged 6–9 weeks, obtained from Guangdong Medical Laboratory Animal Center (Guangzhou, China), were used in the present study. The mice were housed in groups of 4 per cage under standard conditions (temperature at 22–26 °C, relative humidity at 40–60%, and a 12-h light/dark cycle) and provided ad libitum access to water and food. Allocation concealment was strictly implemented during the assignment of mice to control and treatment groups. All animal experimental procedures were approved by the Animal Experimentation Ethics Committee of Guangzhou Medical University (Ethics approval number: N2025-29034) and were performed in accordance with the guidelines of the National Institutes of Health on the care and ethical treatment of animals.

### 2.2 Experimental design

(S)-ketamine (Jiangsu Hengrui Pharmaceutical Co., Ltd., Jiangsu, China) was dissolved in saline for injections. Ketamine was administered as described in our previous study (Luo et al., 2021). Inulin was derived from dahlia, with a polymerization degree of 2–10 oligo fructose (>90% purity; CAS: 9005-80-5; Macklin Biochemical Co., Ltd., Shanghai, China). Mice were randomly divided into the following three groups: (1) ketamine group (Ket-28d): mice were infused intraperitoneally (i.p.) with ketamine (30 mg/kg) once a day for 28 consecutive days and fed a standard diet for 6 weeks. (2) Ketamine with inulin group (Inu + Ket-28d): after injection of ketamine for 28 days, mice were provided drinking water containing inulin (2 g/kg) for 6 weeks as the intervention group; the dose of inulin was based on a previous study (Guo et al., 2021). (3) Vehicle group (Veh): mice were injected i.p. with an equal volume of saline for 28 days and fed a standard diet for 6 weeks. The body weights of all mice were monitored weekly, and food and water intake were recorded daily. The inulin solution was prepared with fresh drinking water according to the daily water intake volume of each mouse. The behavioral tests, molecular experiments, morphological observations, and sequencing analysis were performed after the inulin intervention.

### 2.3 Western blot analysis

The separated hippocampal tissue was homogenized in the Tissue Protein Extraction Reagent (T-PER; Thermo Scientific, Rockford, IL, United States), and protein concentrations were determined as previously described (Luo et al., 2021). Total proteins were separated by 10% sodium dodecyl sulfate (SDS)–polyacrylamide gels and transferred onto polyvinylidene difluoride (PVDF) membranes (Bio-Rad, Hercules, CA, United States). The membranes were blocked for 1 h at room temperature and incubated with primary antibodies and horseradish peroxidase (HRP)-conjugated anti-rabbit secondary antibodies (CST, Boston, United States). Subsequently, the membranes were developed with the SuperSignal West Pico chemiluminescence substrate (Thermo Scientific) and imaged using an imaging system (ChemiDoc XRS+; Bio-Rad).

### 2.4 Enzyme-linked immunosorbent assay

Hippocampal tissues were adequately homogenized in an appropriate volume of cell lysis reagent (Thermo Scientific) as described in our previous study (Xu et al., 2025a). The homogenates were then centrifuged at 14,000 × g for 15 min at 4 °C. The protein-containing supernatants were collected and stored at −80 °C until further experimentation. Blood samples were obtained by enucleating the eyeballs of mice, and serum was separated by centrifugation at 4,000 × g for 15 min at 4 °C, then aliquoted and frozen at −80 °C for further analysis. The levels of BDNF and TrkB in serum and hippocampus were measured using commercial enzyme-linked immunosorbent assay (ELISA) kits (Abnova, Taiwan, China; Novus, Colorado, United States) according to the manufacturer’s protocols. The sensitivities of the BDNF and TrkB assays were 60 pg./mL and 46.88 pg./mL, respectively. All samples and standards were assayed in duplicate and expressed as pg./mL. BDNF and TrkB levels were determined using a microtiter plate reader (iMark; Bio-Rad) set at 450 nm.

### 2.5 Real-time quantitative PCR

Total RNA from hippocampal tissues was extracted using the Mini BEST Universal RNA Extraction Kit (Takara, Shiga, Japan) in accordance with the manufacturer’s instructions. cDNA was generated from total RNA (37 °C for 15 min and 85 °C for 5 s) using PrimeScript™ RT Master Mix (Takara). qRT-PCR was performed using the TB Green® Premix Ex Taq™ II (Takara) and conducted on the Applied Biosystems ViiA 7 Real-time PCR System (Applied Biosystems, Carlsbad, CA, United States). The PCR reaction conditions were as follows: 50 °C for 2 min; 95 °C for 30 s; followed by 40 cycles of 95 °C for 5 s, 56 °C for 30 s, and 72 °C for 1 min.

### 2.6 Immunohistochemistry

Immunohistochemistry was carried out in accordance with a previously described study (Luo et al., 2024). Briefly, mice were anesthetized with amobarbital sodium (50 mg/kg, i.p.) and transcardially perfused with normal saline, followed by 4% paraformaldehyde (PFA). The brains were immediately removed, postfixed in 4% PFA overnight, and then transferred into 20 and 30% sucrose dissolved in phosphate-buffered saline for dehydration, respectively. Hippocampal tissue was cut coronally into slices at a thickness of 20 μm using a freezing vibratome (CM1950; Leica, Heppenheim, Germany). The sections were blocked with 3% bovine serum albumin and 0.2% Triton X-100 in phosphate-buffered saline for 1 h at room temperature, followed by overnight incubation with primary antibodies at 4 °C. Then, the slices were probed with the appropriate fluorescence-labeled secondary antibodies for 1 h at 37 °C and coverslipped with ProLong Gold Antifade Reagent (Thermo Scientific). Finally, the sections were imaged using a fluorescence microscope (Axio Imager Z2, Zeiss, Jena, Germany). In addition, colonic tissue was immersed in 4% PFA overnight, dehydrated, embedded in paraffin, and sliced into 5 μm thickness. After dewaxing, rehydrating, and antigen retrieval, the slices were blocked with goat serum for 30 min at 20–24 °C and incubated with primary antibodies overnight at 4 °C. All slices were subsequently incubated with appropriate HRP-conjugated anti-rabbit secondary antibodies for 1 h at 37 °C and with 3,3′-diaminobenzidine (DAB) development solution for 5 min at room temperature. Images were acquired using an Olympus microscope (BX43, Olympus, Aomori, Japan).

### 2.7 Liquid chromatography tandem mass spectrometry

Serum samples were collected as described in our previous study (Xu et al., 2025a). The concentrations of SCFAs in serum, hippocampus, and feces were detected by the Shenzhen Academy of Metrology and Quality Inspection (Shenzhen, China) using liquid chromatography tandem mass spectrometry (LC–MS/MS). Briefly, hippocampal and fecal SCFAs were extracted from 50 mg solid samples with a methanol-containing buffer. Then, ethyldimethylaminopropyl carbodiimide and 3-nitrophenylhydrazine were added to samples of serum, hippocampus, and feces, respectively, and mixed for 20 min at 4 °C. The mixture was centrifuged at 20,000 × g for 5 min at 4 °C, and the supernatant was transferred to the liquid chromatography vial. A standard curve was established using the corresponding standards. Multiple Reaction Monitoring, Analyst 1.6.3, and MultiQuant 3.0.3 software were used for data acquisition and quantitative analysis.

### 2.8 Hematoxylin and eosin staining

The colonic tissue was immediately fixed with 4% PFA, dehydrated, embedded in paraffin, and cut into 5 μm slices. The colonic slices were then hydrated in xylene and stained with hematoxylin and eosin (HE). Finally, the slices were made transparent with xylene and mounted with neutral gum. The histopathological changes of colonic tissue were examined using an Olympus microscope.

### 2.9 Nissl staining

The brain tissue was embedded in paraffin and coronally cut into 5 μm sections. After dewaxing and hydration, the brain sections were stained with 0.1% toluidine blue for 15 min, treated with deionized water, and dehydrated with gradient ethanol. Finally, the slices were made transparent with xylene and sealed with neutral gum. Neuromorphic and pathological changes in hippocampal tissue were observed under a light microscope (Olympus).

### 2.10 16S rRNA sequencing analysis of fecal samples

Fresh feces were collected from mice and packed into 1.5 mL sterile microcentrifuge tubes, which were immediately frozen in dry ice and stored at −80 °C for later analysis. The QIAamp DNA Stool Mini Kit (Qiagen, Dusseldorf, Germany) was used as described by the manufacturer to extract the fecal microbiota DNA. The V4 hypervariable region of 16S rRNA genes of gut microbiota was amplified using universal primer pairs (515 forward primer: 5′-TCGTCGGCAGCG TCAGATGTGTATAAGAGACAGCCTACGGGNGGCWGCAG-3′, 806 reverse primer: 5′-GTCT CGTGGGCTCGGAGATGTGTATAAGAGACAGGATACHVTATCTAATCC-3′). The PCR amplicon of 16S rRNA genes was performed under the following conditions: 94 °C for 5 min; 30 cycles of 94 °C for 30 s, 52 °C for 30 s, and 72 °C for 30 s; a final extension at 72 °C for 10 min, and then held at 4 °C. Fecal 16S rRNA sequencing and analysis were completed by Guangdong Magigene Biotechnology Co., Ltd. (Guangzhou, China) as previously described (Xu et al., 2025a).

### 2.11 PPI test

The acoustic startle reactivity (ASR) and PPI of the startle reflex were assessed as described previously (Luo et al., 2022). Mice were permitted to acclimate to the behavioral room for 1 h prior to the commencement of the experiment. After acclimatization to the background noise (69 dB; 5 min duration), mice were presented with 10 presentations of a startling pulse (120 dB, 40 ms duration) to adapt to the startle stimuli. The protocol of the PPI test consisted of 80 trials, randomly divided into 8 distinct types, presented with an interval of 10–20 s: 10 presentations of no pulse, 10 presentations of a startling pulse alone (120 dB, 40 ms duration), 10 presentations of each prepulse alone (76, 79, and 85 dB; 20 ms duration), and 10 presentations of each prepulse with a startling pulse (76 + 120 dB, 79 + 120 dB, and 85 + 120 dB; 100 ms interval). The PPI percentage was calculated according to the following formula: (ASR amplitude of startling pulse ASR amplitude of a prepulse with a startling pulse)/(ASR amplitude of a startling pulse) × 100 (Luo et al., 2022). The startle response was estimated by determining the average of all the ASR amplitudes of the startling pulse in each group.

### 2.12 Morris water maze test

The spatial learning and memory of mice were measured using the Morris water maze as previously described (Luo et al., 2021). Mice were allowed to adapt to the behavioral room for 1 h before the experiment. The Morris water maze test was performed in a round pool (diameter, 120 cm; height, 50 cm). The pool was filled to a depth of 20 cm with opaque water made from white non-toxic titanium dioxide and was divided into four quadrants. The escape platform (diameter, 8 cm) was submerged 1 cm under the water surface and placed in the center of the second quadrant of the pool. The water and environment temperature were kept at 22 °C. The water maze test composed of two phases: a 5-day spatial acquisition phase followed by a 1-day spatial probe trial. In the spatial acquisition phase, the starting quadrant was pseudo-randomized across the 5 training days. Each mouse was placed into the pool at one of the four positions and trained to seek the platform, completing the four quadrants in sequence. The time spent, termed escape latency, was recorded by a video tracking system (Guangzhou Feidi Biotechnology Co., Ltd., Guangzhou, China). Times exceeding 60 s were recorded as 60 s. On day 6, the platform was removed from the pool, and each mouse was gently placed in the quadrant opposite the original platform position and allowed to swim freely for 60 s. The number of crossings into the target zone and time spent in the target quadrant were analyzed.

### 2.13 Elevated plus maze test

The anxiety-like behaviors of mice were measured in the elevated plus maze test as previously described (Xu et al., 2025b). The elevated plus maze apparatus was a white Plexiglas structure elevated 70 cm above the floor. It contained two relatively open arms and two relatively closed arms surrounded by 15 cm high walls. Mice were moved to the behavioral room for 1 h before testing. Thereafter, a single mouse was placed in the center of the maze facing an open arm and allowed to freely explore the maze for 5 min, with their behaviors being recorded using an EthoVision XT 11.0 video tracking system (Noldus, Wageningen, The Netherlands). The percentage of time spent in the open arms was measured using the following formula: (time spent in open arms/total time spent in all arms) × 100. The percentage of entries into the open arms was calculated as (number of entries into open arms / total number of entries) × 100.

### 2.14 Forced swimming test

The depression-like behaviors of mice were assessed in the forced swimming test as described in our previous study (Xu et al., 2025b). Mice were allowed to adapt to the behavioral room for 1 h prior to testing. Thereafter, mice were gently placed into Plexiglas cylinders (height: 30 cm, diameter: 20 cm), containing 15 cm of fresh tap water, maintained at 23–25 °C. The experiment lasted 6 min, but only the last 4 min were recorded and analyzed using EthoVision XT 11.0 video tracking software (Noldus). The depression-like behaviors of mice were measured by calculating total immobility time.

### 2.15 Tail suspension test

The depression-like behaviors of mice were analyzed in the tail suspension test according to the methods described in a previous study (Cheng et al., 2025). Mice were moved to the behavioral room for 1 h before testing. Subsequently, mice were gently suspended on a horizontal bar using breathable adhesive tape placed about 2 to 3 cm from the tip of their tail. The test lasted for 6 min, during which the behaviors of the mice were recorded using EthoVision XT 11.0 video tracking software (Noldus). The immobility time was used to evaluate the depression-like behaviors of the mice.

### 2.16 Open field test

The locomotor activity and anxiety-like behaviors of mice were evaluated in the open field as described previously (Guo et al., 2021). The experiment was conducted in a white Plexiglas apparatus (50 × 50 × 50 cm) without a lid. The arena was separated into two parts: the center zone (20 × 20 cm, far from the walls) and the peripheral zone (outside the center area). Mice were acclimatized to the behavioral room for 1 h before testing. Mice were then placed in the center of the chamber and allowed to freely explore it for 5 min. Behavioral parameters were recorded and analyzed using EthoVision XT 11.0 video tracking software (Noldus). The locomotor activity was measured by the total distance moved. The anxiety-like behaviors were evaluated by calculating the time spent in the central area and the number of entries into the center zone.

### 2.17 Statistical analysis

Statistical analysis was carried out using IBM SPSS v25.0 software (Armonk, NY, United States). All experimental data were presented as mean ± standard errors of the mean (SEM). The Shapiro–Wilk normality test and Levene’s test were employed to assess the normality and homogeneity of variance of all data, respectively. The escape latency data from the Morris water maze, PPI % data from the PPI test, and body weight data were analyzed using repeated-measures two-way analysis of variance (ANOVA) with Bonferroni’s post-hoc test. Other behavioral parameter data were evaluated using one-way ANOVA with Bonferroni’s post-hoc test. The ELISA, qRT-PCR, Western blot, and immunohistochemistry data for multiple groups were measured using one-way ANOVA, followed by Bonferroni’s post-hoc test. The LC–MS/MS and gut microbiota data were assessed using one-way ANOVA with Bonferroni’s *post-hoc* test or Kruskal–Wallis test with Dunn’s test. Spearman correlation analysis was used to estimate the correlations between gut microbiota and behavioral parameters, SCFA levels, and concentrations of BDNF and TrkB. The Benjamini–Hochberg method was carried out to control the false discovery rate (FDR) for multiple comparisons in Spearman correlation analysis. Statistical significance was defined as *p* < 0.05.

## 3 Results

### 3.1 Inulin intervention improved anxiety-like behaviors and deficits in spatial learning and memory induced by chronic ketamine administration

The changes in body weight may serve as an indicator of the general health of the mice. Therefore, we recorded the body weights of the mice weekly. As shown in Supplementary Figure S1, body weight significantly decreased in mice injected with ketamine for 28 days, and this decrease was alleviated by inulin treatment.

**Figure 1:**
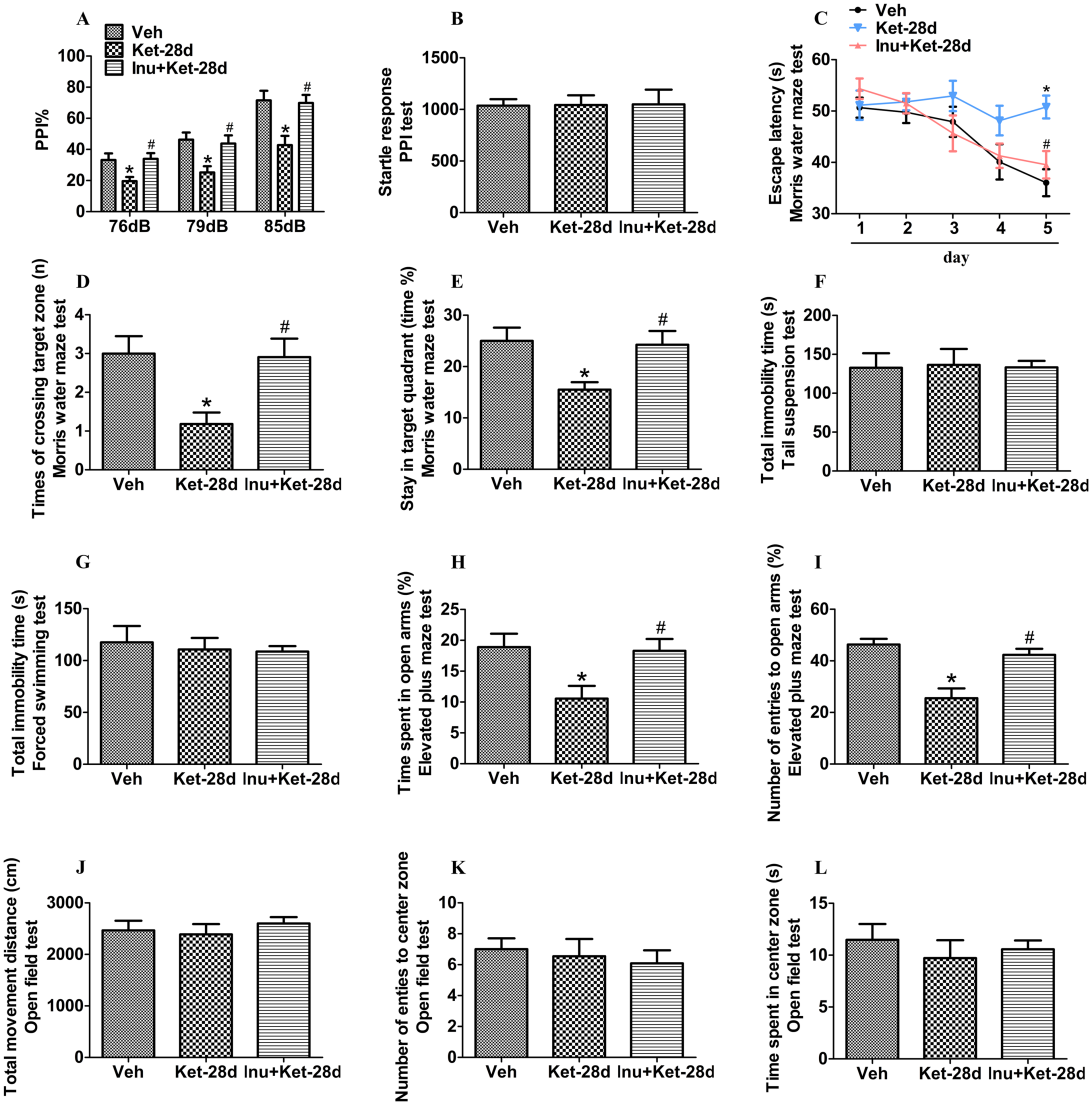
Inulin treatment alleviated anxiety-like behaviors and impairments in spatial learning and memory caused by chronic ketamine exposure. **(A)** A significant reduction in PPI with prepulse intensities of 76 dB, 79 dB, and 85 dB was found in mice injected with ketamine once a day for 28 days, and this reduction was attenuated by inulin administration (*n* = 11). **(B)** There were no significant differences among the three groups in terms of startle amplitude in the PPI test (*n* = 11). **(C)** The latency time to reach the escape platform was significantly increased in mice exposed to ketamine for 28 days, and this increase was ameliorated by inulin intervention (*n* = 11). **(D,E)** The number of times of crossings into the target zone and the amount of time spent in the target quadrant in the probe test were significantly decreased in mice that received ketamine for 28 days; these reductions were alleviated by inulin treatment (*n* = 11). **(F,G)** There were no significant differences among the three groups in total immobility time in both the forced swimming test and the tail suspension test (*n* = 11). **(H,I)** Time spent in open arms and the number of entries into open arms in the elevated plus maze test were significantly reduced in mice exposed to ketamine once a day for 28 days, and these reductions were alleviated by inulin intervention (*n* = 11). **(J–L)** There were no significant differences among the three groups in total movement distance, number of entries into the center zone, and time spent in the center zone in the open field test (*n* = 11). Data are expressed as mean ± SEM; *p < 0.05 vs. the Veh group, ^#^*p* < 0.05 vs. the Ket-28d group. Veh, vehicle; Ket, ketamine; Inu, inulin.

We detected schizophrenia-like behavioral deficits using PPI, the Morris water maze test, the elevated plus maze test, the forced swimming test, the tail suspension test, and the open field test in mice exposed to ketamine for 28 days. As shown in Figure 1A, chronic ketamine exposure for 28 days resulted in a decrease in PPI with prepulse intensities of 76, 79, and 85 dB, suggesting impairments in sensorimotor gating; this decrease was ameliorated by inulin treatment. As differences in startle reflex among groups could confound PPI results, we also measured the startle response. We found no significant differences in startle amplitude in the PPI test among the three groups (Figure 1B). As shown in Figure 1C, a significant increase in the latency time to reach the escape platform was found in mice injected with ketamine for 28 days, indicating deficits in spatial learning acquisition; this increase was alleviated by inulin intervention. Moreover, chronic ketamine exposure for 28 days decreased the number of times of crossings into the target zone and the amount of time spent in the target quadrant in the probe test, indicating impairments in spatial memory maintenance, and these decreases were attenuated by inulin intervention (Figures 1D,E; Supplementary Figures S2A–C). There were no significant differences in total immobility time in both the tail suspension test and the forced swimming test among the three groups (Figures 1F,G). Chronic ketamine exposure for 28 days significantly reduced the time spent in open arms and the number of entries to open arms in the elevated plus maze test, indicating anxiety-like behaviors; these reductions were ameliorated by inulin administration (Figures 1H,I). In addition, there were no significant differences in total movement distance, the number of entries to the center zone, and time spent in the center zone in the open field test between the three groups (Figures 1J–L; Supplementary Figures S2D–F).

### 3.2 Inulin intervention attenuated the downregulation of the BDNF-TrkB-ERK1/2-CREB signaling pathway in the hippocampus induced by chronic ketamine exposure

**Figure 2:**
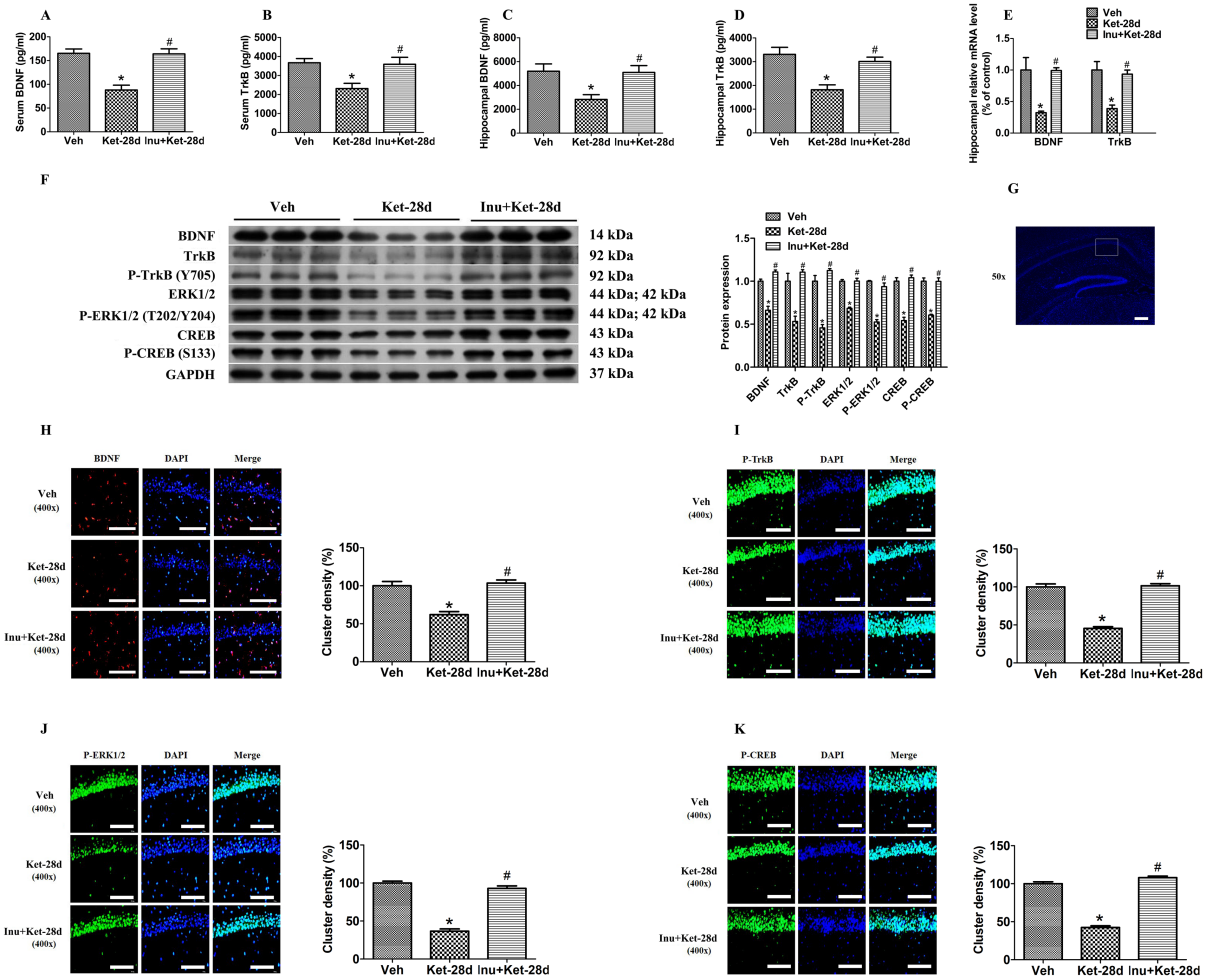
Inulin treatment ameliorated the downregulation of the BDNF-TrkB-ERK1/2-CREB signaling pathway in the hippocampus caused by chronic exposure to ketamine. **(A–D)** Serum and hippocampal BDNF and TrkB levels were significantly decreased in mice injected with ketamine once a day for 28 consecutive days; these reductions were reversed by inulin administration. Quantification was determined by ELISA (*n* = 6). **(E)** Hippocampal mRNA levels of BDNF and TrkB were significantly decreased in mice treated with ketamine once a day for 28 days, and these decreases were alleviated by inulin intervention. Quantification was determined by qRT-PCR (*n* = 6). **(F)** The hippocampal expression of BDNF, TrkB, P-TrkB, ERK1/2, P-ERK1/2, CREB, and P-CREB was significantly reduced in mice exposed to ketamine once a day for 28 days; these decreases were ameliorated by inulin administration. Left: immunoblot analysis of BDNF, TrkB, P-TrkB, ERK1/2, P-ERK1/2, CREB, and P-CREB. Right: quantification of BDNF, TrkB, P-TrkB, ERK1/2, P-ERK1/2, CREB, and P-CREB (*n* = 6). **(G)** Schematic of a hippocampal coronal slice. The white box represents the sampling area for immunostaining analysis. Scale bar: 200 μm. Magnification: 50×. **(H)** The density of BDNF clusters was significantly reduced in mice injected with ketamine for 28 days, and this decrease was alleviated by inulin intervention. Left: immunostaining analysis of BDNF. Scale bar: 100 μm. Magnification: 400×. Right: quantification of BDNF-positive clusters (*n* = 6). **(I)** The density of P-TrkB clusters was significantly decreased in mice exposed to ketamine for 28 days, and this decrease was attenuated by inulin intervention. Left: immunostaining analysis of P-TrkB. Scale bar: 100 μm. Magnification: 400×. Right: quantification of P-TrkB-positive clusters (*n* = 6). **(J)** The density of P-ERK1/2 clusters was significantly reduced in mice infused with ketamine for 28 days, and this reduction was ameliorated by inulin treatment. Left: immunostaining analysis of P-ERK1/2. Scale bar: 100 μm. Magnification: 400×. Right: quantification of P-ERK1/2-positive clusters (*n* = 6). **(K)** The density of P-CREB clusters was significantly reduced in mice given ketamine for 28 days, and this decrease was alleviated by inulin treatment. Left: immunostaining analysis of P-CREB. Scale bar: 100 μm. Magnification: 400×. Right: quantification of P-CREB-positive clusters (*n* = 6). Data are expressed as mean ± SEM; *p < 0.05 vs. the Veh group. ^#^*p* < 0.05 vs. the Ket-28d group. Veh, vehicle; Ket, ketamine; Inu, inulin.

We investigated whether chronic ketamine exposure changed the expressions of hippocampal and serum BDNF and TrkB using ELISA. Our results showed that BDNF and TrkB levels were significantly reduced in both serum and hippocampus in mice injected with ketamine for 28 days, and these reductions were attenuated by inulin treatment (Figures 2A–D). To determine whether the decreased expression of hippocampal proteins BDNF and TrkB was the result of changes in transcription, we performed qRT-PCR to evaluate the changes in mRNA levels. As shown in Figure 2E, the hippocampal mRNA levels of BDNF and TrkB were significantly decreased in mice administered ketamine for 28 days; these decreases were ameliorated by inulin intervention.

We used western blot analysis to examine the downstream signaling molecules of BDNF. As shown in Figure 2F, the hippocampal expression of BDNF, TrkB, P-TrkB, ERK1/2, P-ERK1/2, CREB, and P-CREB was significantly decreased in mice infused with ketamine for 28 days; this decrease was alleviated by inulin intervention.

In addition, immunostaining was used to detect the density of BDNF, P-TrkB, P-ERK1/2, and P-CREB in the hippocampus (Figure 2G). We found that the density of BDNF, P-TrkB, P-ERK1/2, and P-CREB clusters was significantly decreased in the hippocampal CA1 region in mice exposed to ketamine for 28 days, and this reduction was ameliorated by inulin intervention (Figures 2H–K).

### 3.3 Inulin treatment ameliorated neuronal damage and decreases in synaptic protein expression caused by chronic exposure to ketamine

**Figure 3:**
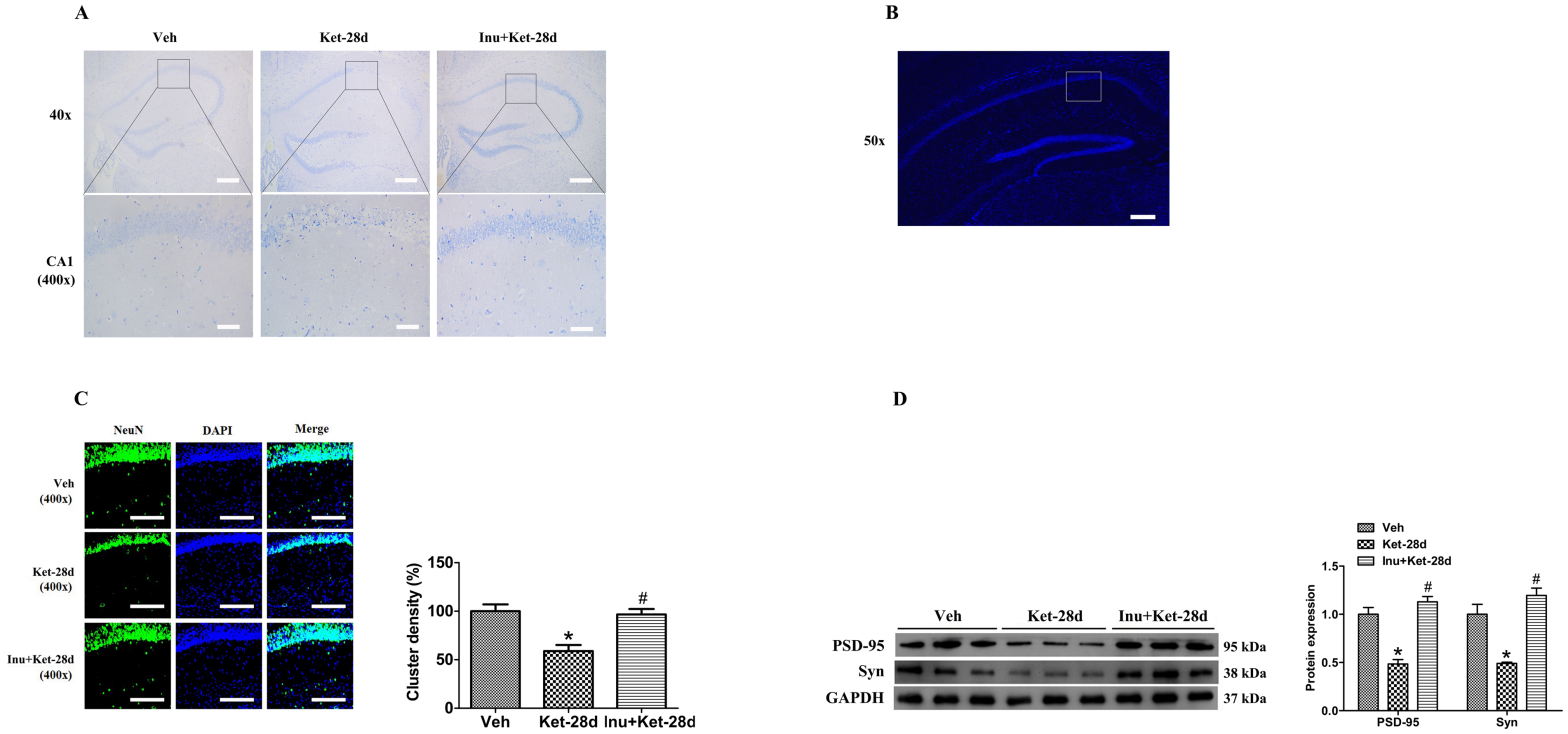
Inulin intervention attenuated neuronal necrosis and reductions in synaptic protein expression induced by chronic ketamine exposure. **(A)** Nissl staining showed that chronic ketamine exposure caused neuronal loss, elevated intercellular space, irregular arrangement of pyramidal neurons, and nuclear condensation with deep staining; these pathological changes were ameliorated by inulin intervention (*n* = 6). Scale bar: 500 μm and 50 μm, respectively. Magnification: 40× and 400×, respectively. **(B)** Schematic of a hippocampal coronal section. The white box represents the sampling area for immunostaining analysis. Scale bar: 200 μm. Magnification: 50×. **(C)** The density of NeuN-positive clusters was significantly reduced in mice exposed to ketamine for 28 days, and this reduction was reversed by inulin intervention. Left: immunostaining analysis of NeuN. Scale bar: 100 μm. Magnification: 400×. Right: quantification of NeuN-positive clusters (*n* = 6). **(D)** Western blot analysis revealed that hippocampal expression of PSD-95 and Syn was significantly decreased in mice exposed to ketamine for 28 days, and these reductions were reversed by inulin treatment. Left: immunoblot analysis of PSD-95 and Syn. Right: quantification of PSD-95 and Syn (*n* = 6). Data are expressed as mean ± SEM; *p < 0.05 vs. the Veh group, ^#^*p* < 0.05 vs. the Ket-28d group. Veh, vehicle; Ket, ketamine; Inu, inulin; NeuN, neuron-specific nuclear protein; PSD-95, postsynaptic density protein-95; Syn, synaptophysin.

We performed Nissl staining and immunostaining to assess the effects of chronic ketamine administration on the physiological and pathological morphology of hippocampal neurons. Nissl staining showed that mice exposed to ketamine for 28 days exhibited neuronal loss, elevated intercellular space, irregular arrangement of pyramidal neurons, and partial nuclear condensation with deep staining; these pathological changes were reversed by inulin treatment (Figure 3A). Immunostaining revealed that the density of neuron-specific nuclear protein (NeuN)-positive clusters was significantly reduced in the hippocampal CA1 region in mice infused with ketamine for 28 days, and this decrease was attenuated by inulin intervention (Figures 3B,C).

To verify whether chronic ketamine exposure caused alterations in synaptic protein expression, we used western blot analysis to detect the levels of PSD-95 and Syn in the hippocampus. Western blot analysis revealed that hippocampal expression of PSD-95 and Syn was significantly reduced in mice given ketamine for 28 days, and this reduction was attenuated by inulin treatment (Figure 3D).

### 3.4 Inulin intervention attenuated impairments in gut barrier integrity and permeability induced by chronic ketamine administration

**Figure 4:**
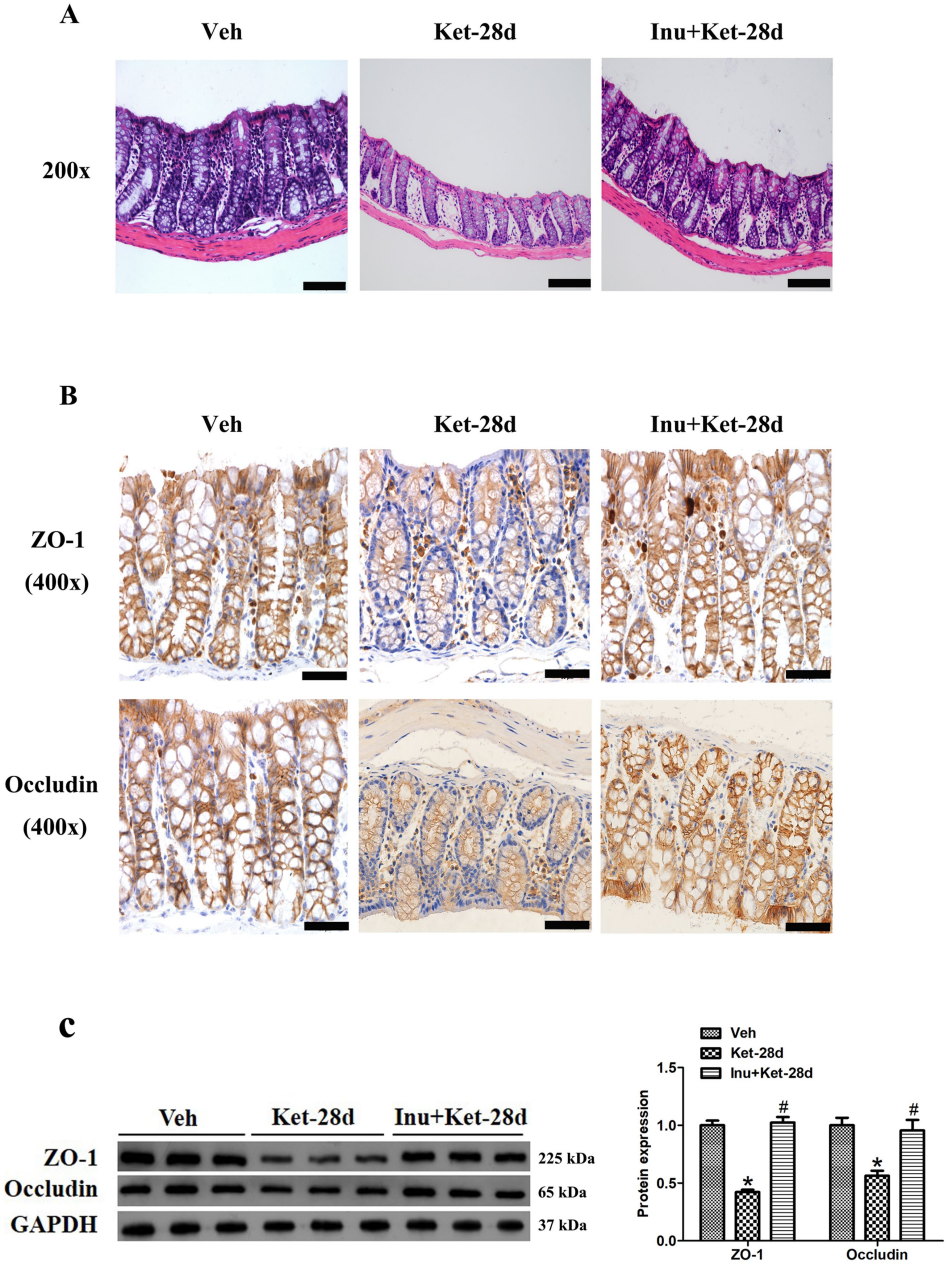
Inulin treatment reversed deficits in gut barrier integrity and permeability caused by chronic ketamine exposure. **(A)** Colonic HE staining revealed that chronic ketamine exposure triggered crypt distortion, loss of certain goblet cells, and thinning of the mucosal layer; these changes in colonic histopathology were attenuated by inulin intervention (*n* = 6). Scale bar: 100 μm. Magnification: 200×. **(B)** Immunostaining analysis showed that colonic expression of ZO-1 and occludin was significantly reduced in mice exposed to ketamine once a day for 28 days; this decrease was ameliorated by inulin intervention (*n* = 6). Scale bar: 50 μm. Magnification: 400×. **(C)** Western blot analysis showed that colonic ZO-1 and occludin levels were significantly reduced in mice given ketamine for 28 days, and this reduction was ameliorated by inulin administration. Left: immunoblot analysis of ZO-1 and occludin. Right: quantification of ZO-1 and occludin (*n* = 6). Data are expressed as mean ± SEM; *p < 0.05 vs. the Veh group, #*p* < 0.05 vs. the Ket-28d group. Veh, vehicle; Ket, ketamine; Inu, inulin.

Next, we examined the colonic morphology and structure using HE staining. Colonic HE staining showed that mice that received a 28-day infusion of ketamine developed a thin mucosal layer, loss of certain goblet cells, and crypt distortion; these alterations in colonic histopathology were alleviated by inulin intervention (Figure 4A). In addition, we used immunostaining and western blot analysis to assess the expression of ZO-1 and Occludin in colonic tissue. As shown in Figures 4B,C, chronic ketamine exposure for 28 days significantly reduced colonic expression of ZO-1 and Occludin, and this reduction was ameliorated by inulin intervention.

### 3.5 Inulin treatment alleviated gut microbiota dysbiosis caused by chronic ketamine exposure

**Figure 5:**
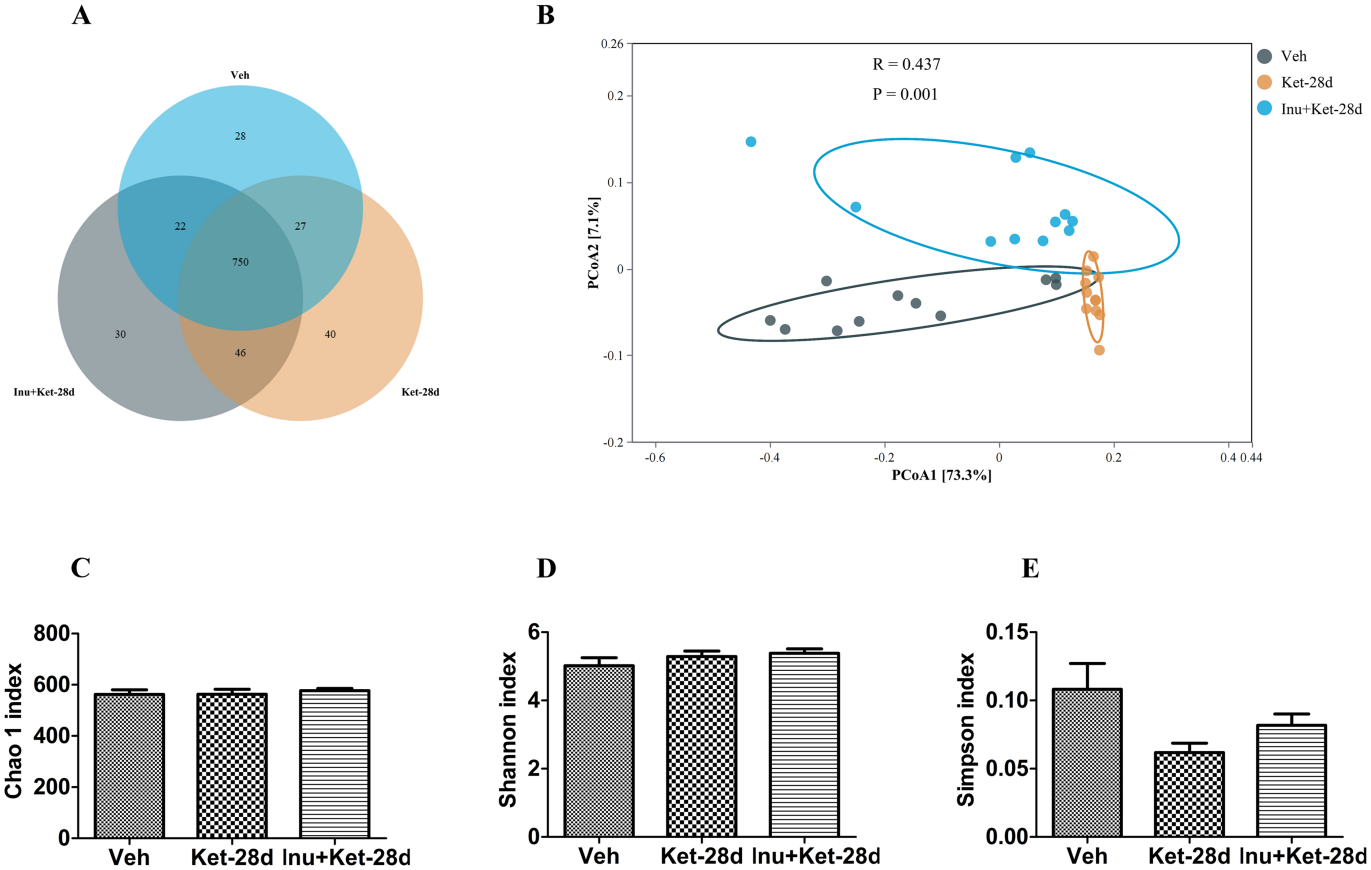
Effects of inulin intervention on α-diversity and β-diversity of gut microbiota in mice subjected to chronic ketamine exposure. **(A)** A Venn diagram revealed that 750 of 943 OTUs were commonly identified in the three groups, while 28, 40, and 30 OTUs were unique to Veh, Ket-28d, and Inu+Ket-28d mice, respectively (*n* = 11). **(B)** PCoA indicated significant differences in β-diversity of gut microbiota among the three groups (*n* = 11). **(C–E)** There were no significant differences among the three groups in α-diversity of gut microbiota assessed by Chao1, Shannon, and Simpson indices (*n* = 11). Data are expressed as mean ± SEM. Veh, vehicle; Ket, ketamine; Inu, inulin; OTUs, operational taxonomic units; PCoA, principal coordinate analysis.

We detected the effect of chronic exposure to ketamine on the α-diversity and β-diversity of gut microbiota using 16S rRNA sequencing and analysis. In total, we obtained 2,712,468 high-quality reads across all samples, which were clustered into 943 operational taxonomic units (OTUs) at 97% sequence similarity. A Venn diagram showed that 750 of 943 OTUs were commonly detected among the three groups, while 28, 40, and 30 OTUs were unique to the Veh, Ket-28d, and Inu + Ket-28d groups, respectively (Figure 5A). Most rarefaction curves reached the saturation plateau, suggesting that the sequencing was sufficient to estimate the species richness of the samples and cover the entire bacterial diversity (Supplementary Figure S3). In addition, we found no significant differences in α-diversity of gut microbiota assessed by Chao 1, Shannon, and Simpson indices among the three groups (Figures 5C–E). Principal coordinate analysis (PCoA) revealed significant differences in β-diversity of gut microbiota among the three groups, indicating alterations in the composition of gut microbiota (Figure 5B).

**Figure 6:**
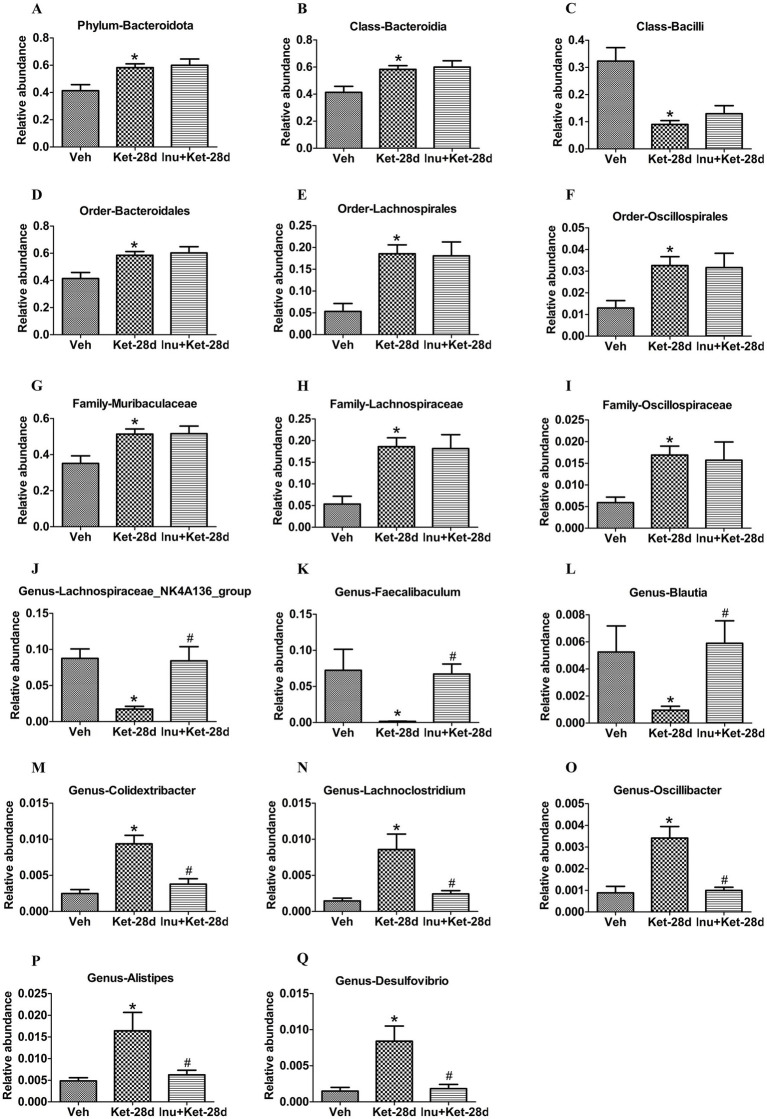
Inulin intervention reshaped gut microbiota dysfunction caused by chronic exposure to ketamine. **(A)** At the phylum level, the abundance of *Bacteroidota* was significantly elevated in mice exposed to ketamine once a day for 28 days (*n* = 11). **(B,C)** At the class level, the abundance of *Bacteroidia* was significantly increased, while the abundance of *Bacilli* was significantly reduced in mice given ketamine once a day for 28 days (*n* = 11). **(D–F)** At the order level, the abundance of *Bacteroidales*, *Lachnospirales*, and *Oscillospirales* was significantly enhanced in mice infused with ketamine once a day for 28 days (*n* = 11). **(G–I)** At the family level, the abundance of *Muribaculaceae*, *Lachnospiraceae*, and *Oscillospiraceae* was significantly increased in mice exposed to ketamine once a day for 28 days (*n* = 11). **(J–Q)** At the genus level, the abundance of *Lachnospiraceae_NK4A136_group*, *Faecalibaculum*, and *Blautia* was significantly reduced, whereas the abundance of *Colidextribacter*, *Lachnoclostridium*, *Oscillibacter*, *Alistipes*, and *Desulfovibrio* was significantly increased in mice injected with ketamine once a day for 28 days; these changes were alleviated by inulin treatment (*n* = 11). Data are expressed as mean ± SEM; *p < 0.05 vs. the Veh group, ^#^*p* < 0.05 vs. the Ket-28d group. Veh, vehicle; Ket, ketamine; Inu, inulin.

We further determined the effects of chronic ketamine exposure on the composition of gut microbiota. At the phylum level, *Bacteroidota* was significantly elevated in mice injected with ketamine for 28 days (Figure 6A). At the class level, *Bacteroidia* was significantly enhanced, while *Bacilli* was significantly reduced in mice exposed to ketamine for 28 days (Figures 6B,C). At the order level, *Bacteroidales*, *Lachnospirales*, and *Oscillospirales* were significantly increased in mice that received ketamine for 28 days (Figures 6D–F). At the family level, *Muribaculaceae*, *Lachnospiraceae*, and *Oscillospiraceae* were significantly elevated in mice given ketamine for 28 days (Figures 6G–I). At the genus level, *Lachnospiraceae_NK4A136_group*, *Faecalibaculum*, and *Blautia* were significantly decreased, whereas *Colidextribacter*, *Lachnoclostridium*, *Oscillibacter*, *Alistipes*, and *Desulfovibrio* were significantly increased in mice administered ketamine for 28 days; these changes were ameliorated by inulin administration (Figures 6J–Q).

**Figure 7:**
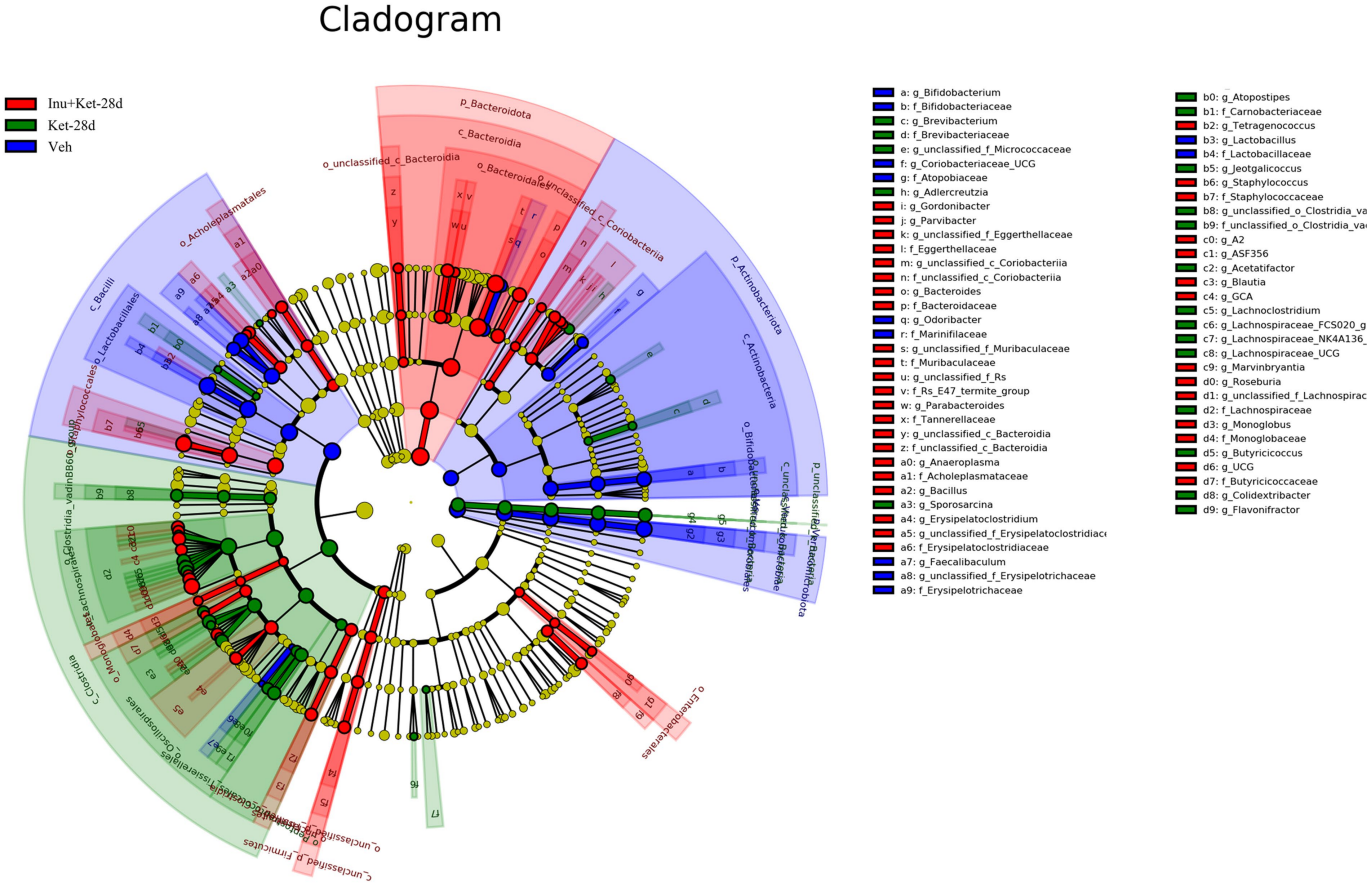
Complete phylogenetic tree based on 16S rRNA sequencing and analysis. The LEfSE cladogram diagram revealed different abundance taxa from the phylum to genus levels among the three groups. Blue plot, Veh group; green plots, Ket-28d group; red plots, Inu+Ket-28d group; yellow plots, non-significant (*n* = 11). Veh, vehicle; Ket, ketamine; Inu, inulin.

**Figure 8:**
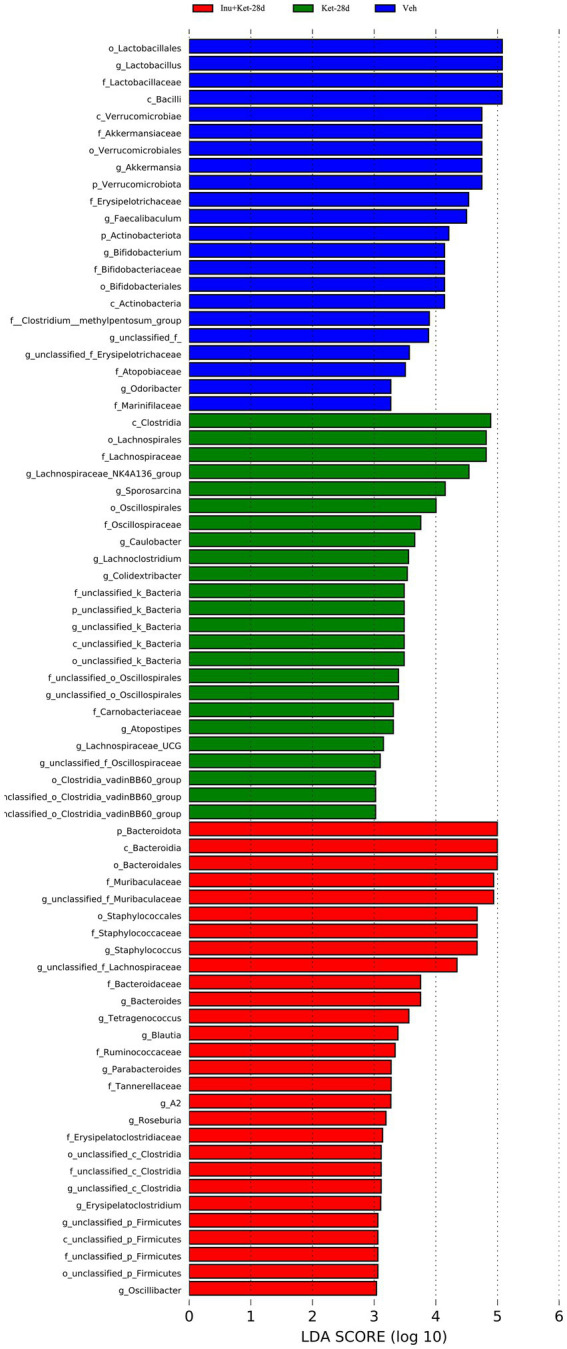
Effects of chronic ketamine exposure on the structure and composition of gut microbiota using LEfSE analysis. Histogram of LDA scores for the different abundance taxa at phylum to genus levels among the three groups. The LDA score was set at >2.0, and the *p* value was < 0.05 (*n* = 11). Veh, vehicle; Ket, ketamine; Inu, inulin; *g*, genus; LDA, linear discriminant analysis; LEfSe, LDA coupled with effect size measurements.

We further evaluated the impacts of chronic ketamine exposure on the abundance of gut microbiota taxa using LEfSE analysis. As shown in Figure 7, the LEfSE cladogram revealed that gut microbiota exhibited significant differences at various taxonomic levels among the three groups. LEfSE analysis identified 74 different abundant taxa from phylum to genus levels among the three groups (LDA > 2). Specifically, a total of 22 bacteria, such as the order *Lactobacillales*, classes *Bacilli*, families *Akkermansiaceae*, and genera *Faecalibaculum*, were significantly enriched in the Veh group (Figure 8). Moreover, a total of 24 bacteria, such as classes *clostridia*, genera *Colidextribacter*, and order *Oscillospirales*, were significantly enriched in the Ket-28d group (Figure 8). Furthermore, a total of 28 bacteria, such as the phylum *Bacteroidota*, families *Muribaculaceae*, and genera *Blautia*, were significantly enriched in the Inu + Ket-28d group (Figure 8).

### 3.6 Inulin intervention alleviated decreases in SCFAs in serum, hippocampus, and feces caused by chronic exposure to ketamine

**Figure 9:**
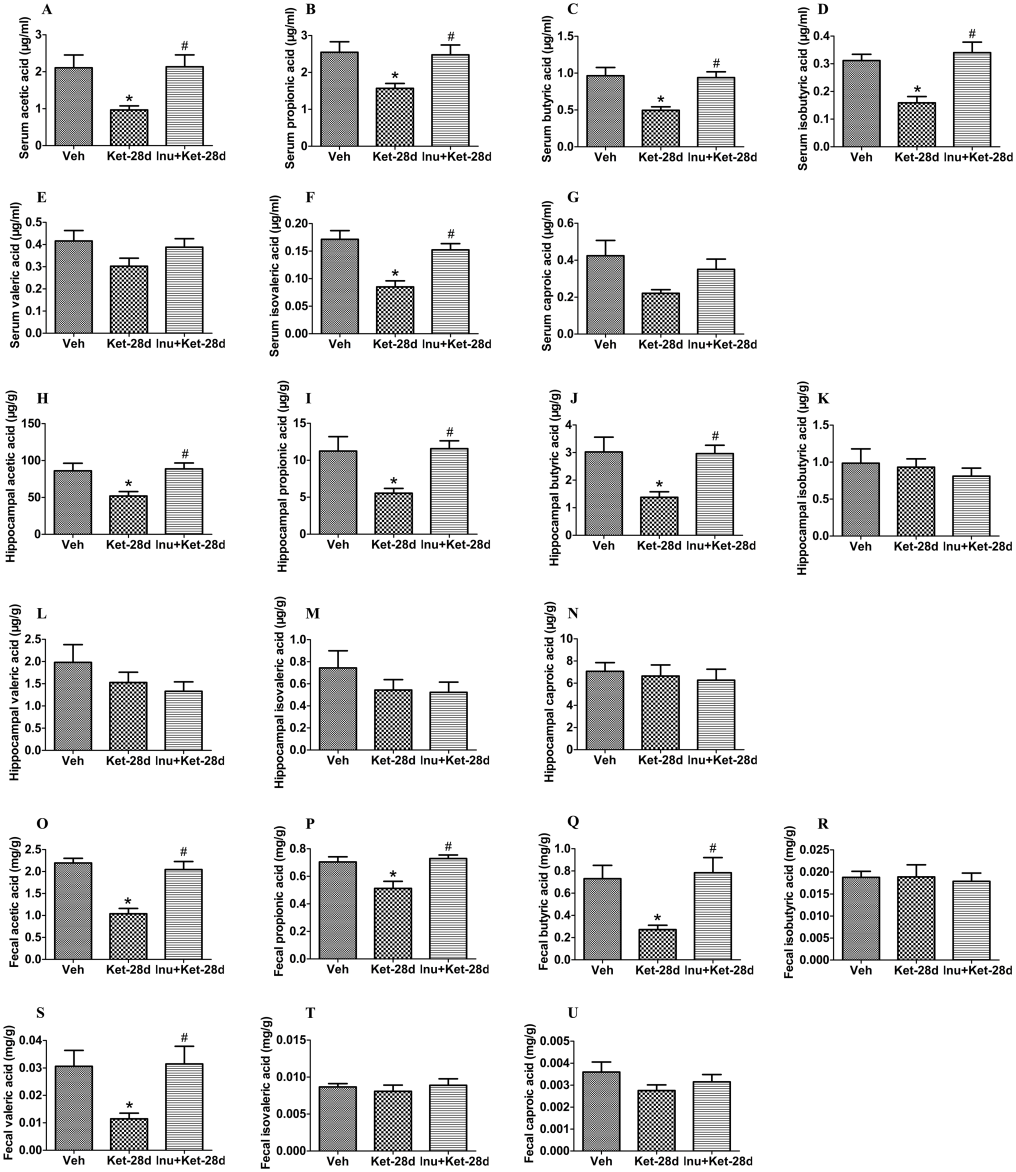
Inulin treatment ameliorated reductions in SCFAs in peripheral serum, hippocampus, and feces induced by chronic ketamine exposure. **(A–D,F)** Serum levels of acetic acid, propionic acid, butyric acid, isobutyric acid, and isovaleric acid were significantly reduced in mice exposed to ketamine for 28 days; these reductions were reversed by inulin intervention. Quantification was determined by LC–MS/MS (*n* = 8). **(E–G)** Serum valeric acid and caproic acid levels were unchanged among the three groups. Quantification was determined by LC–MS/MS (*n* = 8). **(H–J)** Hippocampal levels of acetic acid, propionic acid, and butyric acid were significantly decreased in mice administered ketamine for 28 days, and these decreases were attenuated by inulin intervention. Quantification was determined by LC–MS/MS (*n* = 6). **(K–N)** There were no significant differences in hippocampal levels of isobutyric acid, valeric acid, isovaleric acid, and caproic acid in mice that received ketamine for 28 days. Quantification was determined by LC–MS/MS (*n* = 6). **(O–Q,S)** Fecal levels of acetic acid, propionic acid, butyric acid, and valeric acid were significantly reduced in mice exposed to ketamine for 28 days; these reductions were ameliorated by inulin treatment. Quantification was determined by LC–MS/MS (*n* = 11). **(R,T,U)** Fecal isobutyric acid, isovaleric acid, and caproic acid levels were unchanged among the three groups. Quantification was determined by LC–MS/MS (*n* = 11). Data are expressed as mean ± SEM; *p < 0.05 vs. the Veh group, ^#^*p* < 0.05 vs. the Ket-28d group. Veh, vehicle; Ket, ketamine; Inu, inulin; LC–MS/MS, liquid chromatography tandem mass spectrometry.

We further investigated whether chronic ketamine exposure changed the expression of SCFAs in peripheral serum, hippocampus, and feces using LC–MS/MS. Our results showed that serum levels of acetic acid, propionic acid, butyric acid, isobutyric acid, and isovaleric acid were significantly decreased in mice injected with ketamine for 28 days (Figures 9A–D,F), while serum levels of valeric acid and caproic acid remained unchanged (Figures 9E,G). These decreases in acetic acid, propionic acid, butyric acid, isobutyric acid, and isovaleric acid were alleviated by inulin intervention (Figures 9A–D,F). As shown in Figures 9H–J, the hippocampal levels of acetic acid, propionic acid, and butyric acid were significantly reduced in mice treated with ketamine for 28 days, and these reductions were attenuated by inulin intervention. The concentrations of isobutyric acid, valeric acid, isovaleric acid, and caproic acid in the hippocampus showed no significant differences among the three groups (Figures 9K–N). In addition, fecal levels of acetic acid, propionic acid, butyric acid, and valeric acid were significantly decreased in mice infused with ketamine for 28 days; these decreases were ameliorated by inulin treatment (Figures 9O–Q,S). There were no significant differences in fecal isobutyric acid, isovaleric acid, and caproic acid levels among the three groups (Figures 9R,T,U).

### 3.7 Correlational analysis of the gut microbiota with behavioral parameters, BDNF and TrkB levels, and SCFA levels

Finally, we explored whether gut microbiota was associated with behavioral parameters, BDNF and TrkB levels, and SCFA levels in mice injected with ketamine for 28 consecutive days.

**Figure 10:**
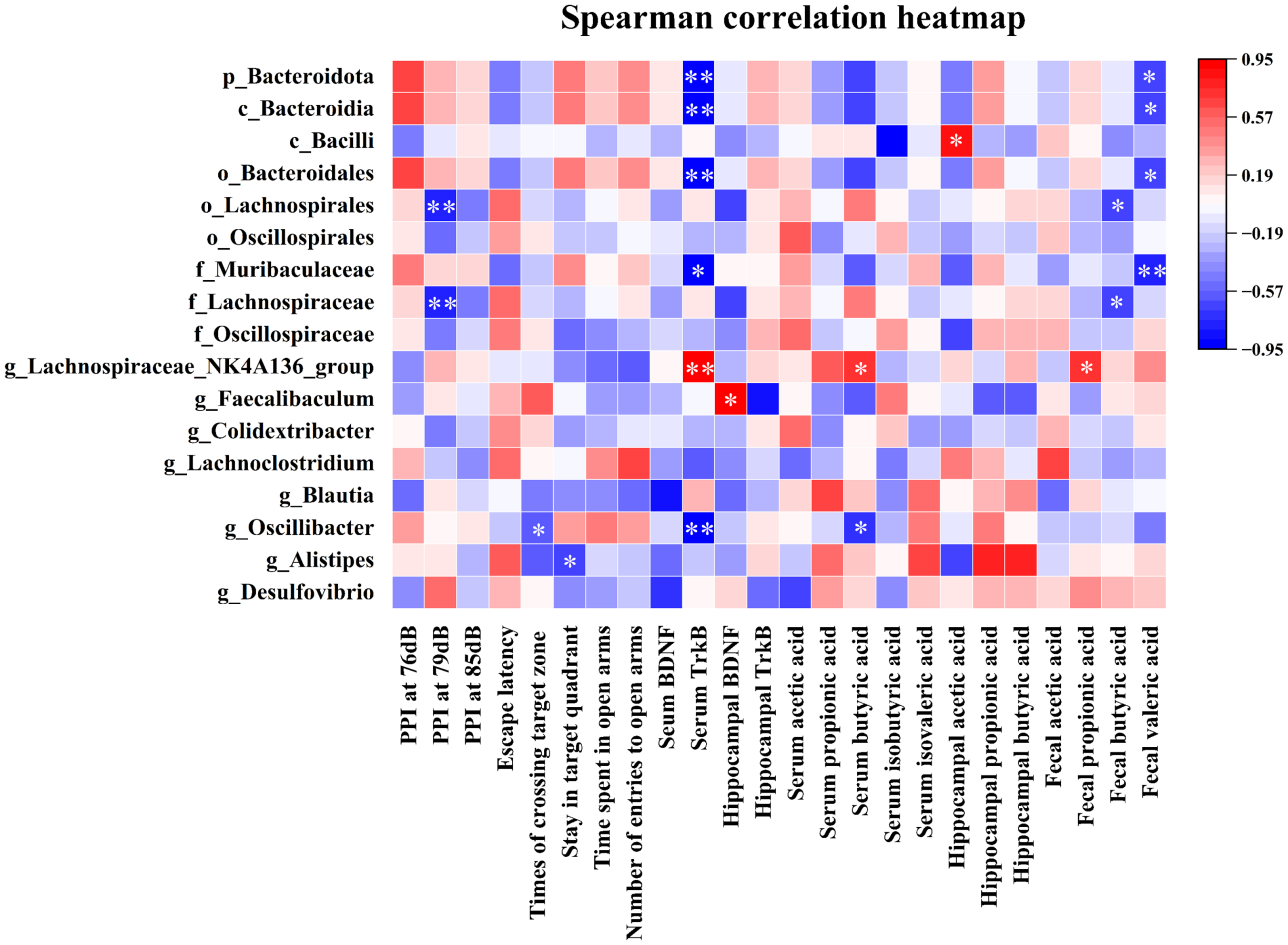
Correlational analysis of the gut microbiota with behavioral parameters, BDNF, TrkB, and SCFA levels. The correlation heatmap showed the associations of the gut microbiota with PPI at 79 dB, number of times of crossings into the target zone, the amount of time spent in the target quadrant, TrkB, BDNF, acetic acid, propionic acid, butyric acid, and valeric acid in mice that received ketamine for 28 days. The red colors indicated positive associations, the blue colors indicated negative correlations, and white indicated no association. *p < 0.05, **p < 0.01. *p*, phylum; *c*, class; *o*, order; *f*, family; *g*, genus.

As shown in Figure 10, *Lachnospirales* at the order level and *Lachnospiraceae* at the family levels were negatively associated with PPI at 79 dB and butyric acid levels in feces. *Oscillibacter* at the genus level was negatively correlated with the number of times of crossings into the target zone and serum TrkB and butyric acid levels. *Alistipes* at the genus level was negatively correlated with the amount of time spent in the target quadrant. *Bacteroidota* at the phylum level, *Bacteroidia* at the class level, *Bacteroidales* at the order level, and *Muribaculaceae* at the family level were negatively associated with serum TrkB levels and fecal valeric acid levels. *Bacilli* at the class level were positively related to hippocampal acetic acid levels. *Lachnospiraceae_NK4A136_group* at the genus level was positively associated with serum TrkB and butyric acid levels and fecal propionic acid levels. Moreover, *Faecalibaculum* at the genus level was positively related to hippocampal BDNF levels.

## 4 Discussion

**Figure 11:**
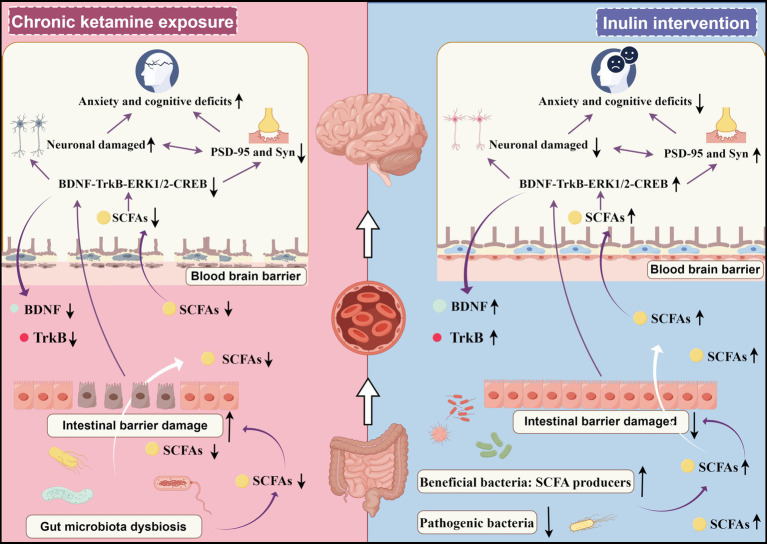
Schematic diagram showing the potential mechanism through which inulin intervention alleviates chronic ketamine-induced anxiety-like behaviors and cognitive deficits. Inulin treatment reverses gut dysbiosis, enhances SCFA levels, improves gut barrier function, and upregulates the BDNF-TrkB-ERK1/2-CREB signaling pathway to reduce neuronal damage and elevate the expression of Syn and PSD-95, which may in turn improve ketamine-induced anxiety-like behaviors, PPI deficits, and spatial learning and memory deficits.

In this study, we found that chronic ketamine exposure for 28 days induced gut microbiota dysregulation, decreased the expression of SCFAs in serum, hippocampus, and feces, increased gut permeability, inhibited the BDNF-TrkB-ERK1/2-CREB signaling pathway, resulted in neuronal damage, and decreased the expression of synaptic proteins Syn and PSD-95, which may lead to anxiety-like behaviors, PPI deficits, and spatial learning and memory deficits (Figure 11). In addition, inulin administration restored gut microbiota dysbiosis by downregulating the abundance of *Colidextribacter*, *Oscillibacter*, *Alistipes*, and *Desulfovibrio,* and upregulating the abundance of *Lachnospiraceae_NK4A136_group*, *Faecalibaculum*, and *Blautia*. This treatment elevated the expression of SCFAs, improved gut barrier integrity, and activated the BDNF-TrkB-ERK1/2-CREB signaling pathway, reducing neuronal damage and increasing the expression of Syn and PSD-95. These changes may, in turn, alleviate chronic ketamine-associated anxiety-like behaviors, PPI impairments, and spatial learning and memory impairments (Figure 11).

### 4.1 Anxiety-like behaviors and spatial learning and memory impairments

PPI is an effective and quantifiable measure for estimating sensorimotor gating, and impairment in sensorimotor gating represents a common psychophysiological feature and a core endophenotype of schizophrenia (Swerdlow et al., 2018). The Morris water maze test is performed to evaluate spatial learning and memory (Luo et al., 2021). The elevated plus maze test is carried out to assess anxiety-like behaviors (Xu et al., 2025b). Previous clinical studies have shown that patients with schizophrenia manifest anxiety-like behaviors and deficits in PPI and visual spatial memory (Swerdlow et al., 2018; Ma and Ma, 2024; Kanchanatawan et al., 2018). In the MK-801 mouse model of schizophrenia, mice injected with MK-801 for 14 days exhibit anxiety-like behaviors and deficits in PPI as well as learning and spatial recognition memory (Guo et al., 2021; Shi et al., 2022). In addition, chronic ketamine abusers also show impairments in PPI and spatial problem solving (Braff et al., 2001; Cheng et al., 2018). Several animal studies have shown that chronic ketamine exposure for 7, 14, and 28 days leads to anxiety-like behaviors, PPI deficits, and spatial learning and memory deficits in mice (Luo et al., 2021; Xu et al., 2025a; Ahmed et al., 2018). Furthermore, inulin treatment ameliorates anxiety-like behaviors and deficits in learning and spatial recognition memory in mice that received MK-801 for 14 days (Guo et al., 2021). Importantly, our previous study showed that inulin intervention improves PPI impairments in mice exposed to ketamine for 14 days (Xu et al., 2025a). In line with these findings, our results revealed that chronic ketamine exposure for 28 days significantly increased the latency time to reach the escape platform but reduced the number of times of crossings into the target zone and the amount of time spent in the target quadrant in the Morris water maze test, decreased PPI, and decreased the time spent in open arms and the number of entries to open arms in the elevated plus maze test, suggesting impairments in spatial learning and memory, deficits in sensorimotor gating, and anxiety-like behaviors, respectively; these alterations were improved by inulin treatment. These findings suggest that inulin intervention could improve schizophrenia-like behaviors that occur after chronic ketamine exposure. Moreover, previous animal studies have demonstrated that chronic exposure to ketamine for 14 and 28 days does not impact locomotor activity in the open field test in mice (Xu et al., 2025a; Luo et al., 2021). Consistent with these results, we found that chronic ketamine exposure for 28 days did not affect locomotor activity in the open field test, suggesting that impairments in spatial learning and memory may not be due to impaired locomotor activity.

### 4.2 Changes in gut microbiota composition

There is some evidence suggesting that gut microbiota dysbiosis may be implicated in the pathophysiology of schizophrenia (Yuan et al., 2025; Okubo et al., 2019; Wu et al., 2026). It has been reported that patients with schizophrenia show abnormal changes in the composition of gut microbiota, which are associated with deficits in visual learning and memory, processing speed, and verbal learning (Zhu et al., 2025a; Zhu et al., 2025b). A clinical study found that *Bifidobacterium breve A-1* treatment for 4 weeks alleviates anxiety and depression symptoms in patients with schizophrenia (Okubo et al., 2019). In the MK-801 mouse model of schizophrenia, mice infused with MK-801 for 14 days exhibit gut microbiota disorders similar to those observed in schizophrenia patients (Guo et al., 2021). In the Poly I: C mouse model of schizophrenia, prenatal Poly I: C exposure induces gut microbiota dysbiosis in male and female offspring during adolescence and adulthood, which is associated with anxiety-like behaviors and PPI impairments (Xu et al., 2025b). Notably, mice receiving gut microbiome from patients with schizophrenia exhibit schizophrenia-like behavioral deficits, including hyperactivity, anxiety-like behaviors, social interaction deficits, and memory impairments (Wei et al., 2024). In addition, voluntary exercise alleviates autism-like behaviors in rats by reshaping gut microbiota dysbiosis (Zhong et al., 2026). However, the effect of gut dysbiosis on chronic ketamine-induced schizophrenia-like behaviors remains unclear. Only a few studies have found that mice injected with ketamine for 5 and 14 days show gut microbiota dysregulation, which may be associated with associative PPI and memory deficits (Xie et al., 2024; Xu et al., 2025a). In this study, we found that chronic ketamine exposure for 28 days did not significantly change the α-diversity of gut microbiota, which is consistent with a recent study (McGuinness et al., 2022). Additionally, we found that chronic exposure to ketamine for 28 days altered the β-diversity of gut microbiota, indicating changes in the composition of gut microbiota, and these alterations resembled those found in schizophrenia patients. Specifically, at the genus level, the abundances of *Lachnospiraceae_NK4A136_group*, *Faecalibaculum*, and *Blautia* were significantly reduced, while the abundances of *Colidextribacter*, *Oscillibacter*, *Alistipes*, and *Desulfovibrio* were significantly increased in mice exposed to ketamine for 28 days. Our correlation analysis revealed that *Lachnospirales* at the order level and *Lachnospiraceae* at the family level were negatively correlated with PPI at 79 dB. *Oscillibacter* at the genus level was negatively associated with the number of times of crossings into the target zone. *Alistipes* at the genus level was negatively associated with the amount of time spent in the target quadrant. Consistent with our findings, several recent studies have indicated that the *Blautia* genus is significantly decreased and the *Colidextribacter* and *Alistipes* genera are significantly increased in schizophrenia patients and the ketamine mouse model of schizophrenia (Xu et al., 2025a; Yuan et al., 2025; Deng et al., 2022). The genus *Oscillibacter* is significantly enhanced in patients with schizophrenia and is negatively correlated with impairments in logical memory, visual learning, and working memory (Ma et al., 2022). The abundance of the genus *Desulfovibrio* is significantly increased and negatively associated with verbal learning index scores in schizophrenia patients, while the abundance of the genus *Faecalibacterium* is significantly reduced and positively associated with visual learning index scores in patients with schizophrenia (Li H. et al., 2024). Therefore, gut microbiota dysbiosis caused by chronic ketamine exposure may account for anxiety-like behaviors, deficits in PPI, and deficits in spatial learning and memory that occur after chronic exposure to ketamine.

It has been reported that the *Lachnospiraceae_NK4A136_group* genus could produce SCFAs by fermentation and metabolism of dietary fiber, thereby maintaining the integrity and permeability of the gut barrier and exerting immunomodulatory effects (Yang P. et al., 2024). The *Alistipes* genus could catabolize tryptophan into indole and reduce the availability of circulating 5-hydroxytryptamine, modulating the balance of neurotransmitters in the CNS, and thus affecting brain functions and behaviors (Yang P. et al., 2024; Parker et al., 2020). A recent study found that the abundance of the *Lachnospiraceae_NK4A136_group* genus is significantly decreased while the abundance of the Alistipes genus is significantly increased in acute colitis mice; these changes are reversed by inulin treatment (Wang et al., 2019). The *Faecalibaculum* genus, a part of the *Erysipelotrichaceae* family, has been reported to convert polysaccharides into SCFAs and improve cognitive function by reducing immune inflammatory responses (Xu et al., 2025a; D'Amato et al., 2020). A previous animal study indicated that mice transplanted with gut microbiota from aged donors show decreases in fecal SCFA levels and the abundance of *Faecalibaculum*, which may be implicated in synaptic plasticity impairments and spatial learning and memory deficits (D'Amato et al., 2020). The *Blautia* genus, a core member of the gut microbiota in both mice and humans, is an important SCFA producer and could reduce inflammatory response by upregulating regulatory T cells and inhibiting the expression of proinflammatory cytokines (Zhang et al., 2024). An animal study demonstrated that inulin treatment alleviates hepatic steatosis in rats partly through increasing the abundance of the *Blautia* genus and fecal SCFA levels (Yang et al., 2023). Previous studies have shown that the *Colidextribacter* genus is involved in cellular oxidative stress response and regulations of inflammation markers (Yang Z. et al., 2024; Duan et al., 2021). Meanwhile, reduced abundance of *Colidextribacter* may help mitigate the severity of peripheral inflammation and neuroinflammation (Yang P. et al., 2024). As Gram-positive bacteria, the *Oscillibacter* genus has a close relationship with neuroinflammation in Alzheimer’s disease rats, and decreased abundance of the *Oscillibacter* genus attenuates cognitive deficits and enhances learning and memory abilities (Yang Z. et al., 2024; Liu et al., 2025). The *Oscillibacter* genus is significantly increased in high-fat diet mice, and this increase is ameliorated by inulin intervention (Zhang et al., 2019). The increased abundance of the *Desulfovibrio* genus could disrupt the integrity and permeability of the gut mucosal barrier and exacerbate the intestinal inflammatory response (Guo et al., 2021). Meanwhile, its metabolites, lipopolysaccharides and H2S, may facilitate the aggregation of α-synuclein (Mohammadi et al., 2025). In the present study, our results revealed that inulin treatment restored gut microbiota dysbiosis in mice exposed to ketamine by downregulating potential pathogenic bacteria (*Colidextribacter*, *Oscillibacter*, *Alistipes*, and *Desulfovibrio*) and upregulating beneficial bacteria (*Lachnospiraceae_NK4A136_group*, *Faecalibaculum*, and *Blautia*). These findings indicate that inulin may attenuate chronic ketamine-induced schizophrenia-like behaviors by restoring gut microbiota dysbiosis occurring after chronic ketamine exposure.

### 4.3 Decreases in SCFA concentrations

Previous studies have found that certain gut microbiota have physiological characteristics of synthesizing and secreting SCFAs (Yang et al., 2022; Lu et al., 2022; Yang P. et al., 2024). For instance, the *Lachnospiraceae_NK4A136_group*, *Faecalibaculum*, and *Blautia* genus are recognized as important SCFA producers and can synthesize them through the fermentation of dietary fiber and polysaccharides (Yang P. et al., 2024; Xu et al., 2025a; Zhang et al., 2024). A previous study has shown that patients with schizophrenia exhibit decreased abundance of the genera *Faecalibacterium* and *Blautia*, which may correlate with reduced expression of fecal SCFAs (Deng et al., 2022). In a mouse model of Alzheimer’s disease, the genera *Turicibacter*, *Roseburia*, and *Blautia* are positively associated with fecal acetate, propionate, and butyrate levels (Yang et al., 2022). In this study, we found for the first time that chronic ketamine exposure for 28 days reduced the abundance of SCFA producers (*Lachnospiraceae_NK4A136_group*, *Faecalibaculum*, and *Blautia*). Our correlation analysis showed that *Bacteroidota* at the phylum level, *Bacteroidia* at the class level, *Bacteroidales* at order level, and *Muribaculaceae* at the family level were negatively correlated with fecal valeric acid levels. *Bacilli* at the class level were positively associated with hippocampal acetic acid levels. *Lachnospirales* at the order level and *Lachnospiraceae* at the family level were negatively correlated with fecal butyric acid levels. *Lachnospiraceae_NK4A136_group* at the genus level was positively associated with serum butyric acid and fecal propionic acid levels. In addition, *Oscillibacter* at the genus level showed a negative association with serum butyric acid levels. Taken together, these findings show that gut microbiota play an important role in modulating SCFA levels.

It has been reported that SCFAs can directly cross the BBB to reach the brain (O'Riordan et al., 2022; Ju et al., 2023). A previous study found that butyrate levels are significantly reduced in the brain tissue of mice with vascular dementia compared to healthy mice (Liu et al., 2015). In addition, a recent study detected SCFA levels (including acetic acid, propionic acid, and butyric acid) in the hypothalamic paraventricular nucleus in rats, and reduced butyric acid is associated with oxidative stress and neuroinflammation (Chao et al., 2022). These findings indicate that SCFAs can cross the BBB and persist within brain parenchyma. Accumulating evidence suggests that reduced SCFA levels are involved in the pathogenesis of schizophrenia (Deng et al., 2022; Peng et al., 2022). Two previous clinical studies found that patients with schizophrenia show reductions in valeric acid and caproic acid levels in serum, as well as decreases in acetic acid, propionic acid, butyric acid, isovaleric acid, and isohexanoic acid levels in feces, and that lower caproic acid levels in serum are positively related to immediate memory (Deng et al., 2022; Peng et al., 2022). A recent animal study demonstrated that the levels of acetic acid, propionic acid, and butyric acid are decreased in the feces of mice on high-methionine diets, which may be correlated with anxiety-like behaviors and impaired spatial learning and memory (Yang et al., 2022). Administration of butyric acid significantly alleviates anxiety-like behaviors and spatial learning and memory deficits in mice exposed to chronic lead (Li Y. et al., 2024). Only one study has demonstrated that chronic ketamine exposure for 6 weeks reduces the fecal succinic acid levels in ovariectomized mice (Wan et al., 2022). In this study, we found for the first time that chronic ketamine exposure for 28 days significantly reduced acetic acid, propionic acid, butyric acid, isobutyric acid, and isovaleric acid levels in serum, decreased acetic acid, propionic acid, and butyric acid levels in the hippocampus, and reduced acetic acid, propionic acid, butyric acid, and valeric acid levels in feces. Together, these findings suggest that decreased SCFA levels caused by gut microbiota dysbiosis may be responsible for anxiety-like behaviors, impairments in PPI, and deficits in spatial learning and memory that occur after chronic ketamine treatment. Intriguingly, inulin intervention ameliorates diphenoxylate-induced anxiety-like behaviors by modulating the composition of gut microbiota and increasing fecal SCFA levels (Zou et al., 2024). Moreover, inulin treatment could attenuate anxiety-like behaviors and impaired spatial learning and memory in obese mice by restructuring gut microbiota and elevating the formation of SCFAs in feces (Liu et al., 2020). In this study, we found that inulin treatment alleviated the decreases in acetic acid, propionic acid, and butyric acid levels in serum, hippocampus, and feces, as well as isobutyric acid and isovaleric acid levels in serum, and valeric acid levels in feces. These results indicate that inulin may alleviate chronic ketamine-induced schizophrenia-like behaviors by upregulating gut dysbiosis-induced decreases in SCFA levels that occur following chronic ketamine exposure.

### 4.4 Downregulations of the BDNF-TrkB-ERK1/2-CREB signaling pathway

The expression of BDNF is largely regulated by gut microbiota and its metabolite SCFAs (Suda and Matsuda, 2022; Yang P. et al., 2024). A previous study revealed that the levels of BDNF and TrkB are significantly decreased in the hippocampus and cortex of antibiotic-treated mice compared to control mice, and these decreases are reversed to normal levels after bacteria recolonization (Sun et al., 2023). An animal study found that SCFA intervention can accelerate the expression of hippocampal BDNF by inhibiting the activity of histone deacetylase (Suda and Matsuda, 2022). In the MK-801 mouse model of schizophrenia, the genera *Lactobacillus*, *Parasutterella*, and *Alistipes* are positively correlated with hippocampal BDNF levels, while the genera *Alloprevotella, Lachnospiraceae_NK4A136_group*, and *Akkermansia* are negatively correlated with hippocampal BDNF levels (Guo et al., 2021; Yang P. et al., 2024). However, the association of gut microbiota and the metabolite SCFAs with chronic ketamine-induced BDNF levels is still unclear. In the present study, we found that *Bacteroidota* at the phylum level, *Bacteroidia* at the class level, *Bacteroidales* at the order level, *Muribaculaceae* at the family level, and *Oscillibacter* at the genus level are negatively correlated with serum TrkB levels. *Lachnospiraceae_NK4A136_group* at the genus level is positively correlated with serum TrkB levels. Furthermore, *Faecalibaculum* at the genus level is positively associated with hippocampal BDNF levels. These findings support the important role of gut microbiota and its metabolites SCFAs in regulating the expression of BDNF.

It has been reported that the BDNF-TrkB pathway plays a crucial role in modulating hippocampal neurogenesis and synaptic function, and that decreases in BDNF may be implicated in schizophrenia through dysfunction of synaptic transmission and plasticity, leading to psychotic and cognitive symptoms (Nieto et al., 2013; Shi et al., 2022). In addition, the binding of BDNF to TrkB activates downstream signaling molecules such as ERK1/2, CREB, and NF-κB, which are important for the transcription of many neuronal genes correlated with synaptic structure and function, synaptic integrity, synaptic plasticity, cognitive function, and behaviors (Luo et al., 2021; Guo et al., 2015). Two previous clinical studies have found that patients with schizophrenia exhibit decreased BDNF levels in serum and hippocampus, and that this decrease in serum is correlated with positive and negative symptoms, as well as cognitive deficits in attention, perceptual-motor skills, processing speed, and memory (Dwivedi et al., 2003; Carlino et al., 2011). In an MK-801 mouse model of schizophrenia, BDNF intervention significantly alleviates neuronal necrosis in the hippocampus and improves anxiety-like behaviors, impaired PPI, and short-term memory ability in mice (Shi et al., 2022). An animal study has reported that rats exposed to MK-801 for 14 days show decreased BDNF-ERK1/2-CREB signaling in the hippocampus, which is associated with spatial learning and memory impairments (Guo et al., 2015). In addition, a significant decrease in serum BDNF levels is observed in chronic ketamine users (Ke et al., 2014). Previous animal studies have indicated that chronic ketamine exposure for 5 and 7 days reduces hippocampal BDNF levels in rats, and that this decrease may be related to anxiety-like behaviors and impairments in PPI and spatial working memory (Ahmed et al., 2018; Célia Moreira Borella et al., 2016). Consistent with these findings, we found that chronic ketamine exposure for 28 days decreased serum BDNF and TrkB levels and reduced hippocampal expression of BDNF, TrkB, ERK1/2, and CREB, suggesting impaired BDNF-TrkB-ERK1/2-CREB signaling. Taken together, downregulation of the BDNF-TrkB-ERK1/2-CREB pathway mediated by gut dysbiosis may be implicated in anxiety-like behaviors, impairments in PPI, and deficits in spatial learning and memory that occur following chronic ketamine exposure. Notably, inulin intervention improves anxiety-like behaviors and impairments in learning and spatial recognition memory by modulating gut microbiota and elevating brain BDNF levels in mice injected with MK-801 for 14 days (Guo et al., 2021). In addition, inulin administration attenuates spatial learning and memory deficits in diabetic rats by restructuring gut microbiota and enhancing the BDNF-TrkB signaling pathway (Morshedi et al., 2020). In this study, we found that inulin treatment ameliorated reductions in BDNF and TrkB levels in serum, as well as decreases in BDNF, TrkB, ERK1/2, and CREB levels in the hippocampus. These results indicate that inulin may ameliorate chronic ketamine-induced schizophrenia-like behavioral deficits by upregulating gut dysbiosis-induced inhibition of the BDNF-TrkB-ERK1/2-CREB signaling pathway that occurs following chronic ketamine administration.

The Syn and PSD-95, important presynaptic and postsynaptic markers, respectively, play crucial roles in maintaining and regulating synaptic strength, synaptic plasticity, cognitive function, and emotion (Luo et al., 2021; Yang P. et al., 2024). Reduced levels of PSD-95 and Syn contribute to impaired synaptic connections and dysfunction, leading to aberrant neural network activity and increased neuronal vulnerability (Han et al., 2023; Yuan and Wang, 2025). Furthermore, severe neuronal damage and necrosis can downregulate the expression of PSD-95 and Syn, resulting in the disruption of synaptic structure and function (Han et al., 2023; Yuan and Wang, 2025). Previous studies have found that chronic restraint stress downregulates the hippocampal expression of the BDNF-TrkB-ERK1/2-CREB pathway in mice by inducing gut microbiota dysbiosis and decreasing serum and fecal SCFA levels, which may be related to neuronal necrosis and reductions in hippocampal PSD-95 and Syn levels (Yao et al., 2024; Du et al., 2023; Wang et al., 2023; Morshedi et al., 2020). A recent clinical study indicated that schizophrenia patients exhibit reduced levels of PSD-95 and Syn in the olfactory bulb (Egbujo et al., 2015). In a mouse model of schizophrenia, the mice show significant decreases in hippocampal Syn and PSD-95 levels, which may be correlated with anxiety-like behaviors and deficits in PPI and social interaction (Yang P. et al., 2024; Dutra-Tavares et al., 2023). Our previous study found that chronic ketamine exposure reduces the hippocampal expression of synaptic proteins Syn and PSD-95, impairs synaptic transmission and long-term potentiation (LTP), thereby leading to spatial learning and memory deficits in mice (Luo et al., 2021). Moreover, chronic ketamine exposure for 14 days induces neuronal necrosis in mice (Xu et al., 2025a). Consistent with these findings, we found that chronic ketamine exposure for 28 days reduced Syn and PSD-95 levels in the hippocampus, caused neuronal damage, and reduced the expression of NeuN in the hippocampus, suggesting impaired synaptic structure and function and neuronal necrosis. Taken together, these findings suggest that downregulation of the BDNF-TrkB-ERK1/2-CREB signaling pathway caused by gut dysbiosis contributes to decreases in Syn and PSD-95 levels and neuronal damage, thus leading to anxiety-like behaviors, deficits in PPI, and deficits in spatial learning and memory that occur following chronic ketamine exposure. Strikingly, a previous animal study found that inulin treatment alleviates anxiety and spatial learning and memory deficits in obese mice by reshaping gut microbiota, increasing fecal SCFA levels, and elevating PSD-95 levels in the cortex (Liu et al., 2020). Inulin administration also restores gut dysbiosis and improves hippocampal neuronal necrosis in mice, thereby ameliorating anxiety-like behaviors and deficits in learning and spatial recognition memory (Guo et al., 2021). In this study, we found that inulin intervention alleviated reduced Syn and PSD-95 levels and neuronal damage in the hippocampus. These results indicate that inulin treatment reverses the downregulation of the BDNF-TrkB-ERK1/2-CREB signaling pathway mediated by gut dysbiosis, thereby increasing Syn and PSD-95 levels, improving neuronal damage, and thus attenuating chronic ketamine-induced schizophrenia-like behaviors.

### 4.5 Impairments in intestinal barrier integrity and permeability

Gut microbiota and its metabolites, SCFAs, are involved in maintaining the integrity and permeability of the intestinal mucosal barrier (Guo et al., 2021; Suda and Matsuda, 2022). The tight junction proteins ZO-1 and Occludin are crucial for building and maintaining the gut mucosal barrier (Maes et al., 2019). Gut microbiota can modulate the expression of tight junction proteins ZO-1 and Occludin by changing SCFA levels, thereby affecting the structure and function of the gut barrier (Maqsood and Stone, 2016). It has been reported that BDNF is primarily synthesized and secreted by neuronal cells in the CNS (Nieto et al., 2013). Intestinal barrier damage, manifested as decreased levels of tight junction proteins, can cause leaky gut, leading to the translocation of pathogenic bacteria and harmful metabolites from the intestine into systemic circulation, which induces peripheral and central inflammatory responses, resulting in neuronal injury and apoptosis, and thus leading to a reduction in brain BDNF levels (Xu et al., 2025a; Guo et al., 2021; Yang P. et al., 2024). The study found that chronic unpredictable mild stress causes intestinal barrier damage in mice by inducing gut microbiota disorder and reducing SCFA levels, which may be associated with the reduced BDNF levels in the hippocampus (Wang et al., 2023). In addition, SCFAs have been reported to directly cross the gut barrier and BBB to facilitate BDNF expression in the hippocampus by inhibiting histone deacetylase, thus affecting neuronal development and synaptic functions (Suda and Matsuda, 2022; D'Amato et al., 2020). These findings indicate that intestinal barrier damage mediated by gut dysbiosis may contribute to decreased brain BDNF levels.

Clinical studies have found that patients with schizophrenia exhibit decreased expression of intestinal tight junction proteins (Maes et al., 2019). In the MK-801 mouse model of schizophrenia, chronic MK-801 administration for 14 days reduces ZO-1 and Occludin levels in the small intestine, which are correlated with anxiety-like behaviors and deficits in learning and spatial recognition memory (Guo et al., 2021). In addition, mice injected with ketamine for 14 days show decreased colonic ZO-1 and Occludin levels, which are involved in ketamine-induced PPI and recognition memory deficits (Xu et al., 2025a). Interestingly, inulin intervention reverses the decrease in the expression of colonic ZO-1 and Occludin and ameliorates neuronal necrosis in the hippocampus by restoring gut microbiota homeostasis, thereby improving impairments in PPI and recognition memory in mice exposed to ketamine (Xu et al., 2025a). Consistent with these findings, we found that chronic ketamine exposure for 28 days decreased ZO-1 and Occludin levels in the colon, suggesting increased intestinal barrier permeability and impaired gut integrity; these changes were alleviated by inulin intervention. These results indicate that chronic ketamine exposure impairs the integrity of the gut barrier and elevates gut permeability by disrupting gut microbiota and decreasing SCFA levels, which may in turn downregulate the BDNF-TrkB-ERK1/2-CREB signaling pathway, leading to anxiety-like behaviors, PPI deficits, and spatial memory deficits. In addition, inulin may improve gut barrier integrity and reduce gut permeability by restoring gut dysbiosis and increasing SCFA levels, thereby upregulating the BDNF-TrkB-ERK1/2-CREB signaling pathway, and thus ameliorating chronic ketamine-induced schizophrenia-like behavioral deficits.

### 4.6 Limitations

Our study has some limitations. First, given the diverse number and types of gut microbiota, it is difficult to identify causal relationships between specific microbiota and schizophrenia. Further studies, such as the transplantation of gut microbiota from ketamine-treated mice to normal mice, are necessary to investigate the precise mechanism by which gut microbiota contributes to chronic ketamine-induced anxiety-like behaviors, deficits in PPI, and deficits in spatial learning and memory. Second, we lacked a group with only inulin intervention in our experimental design, which may influence our exploration of the intervention effects of inulin under ketamine-induced pathological conditions. Inulin has various biological functions, such as reshaping gut microbiota and modulating immune, metabolic, and endocrine pathways (Zou et al., 2024; Qin et al., 2023; Bao et al., 2020). Extensive evidence suggests that inulin exerts beneficial modulatory effects by reversing abnormal compositions of gut microbiota (Zou et al., 2024; Yang et al., 2023; Qin et al., 2023). Furthermore, as a prebiotic, inulin may exert effects through pathways other than regulating gut microbiota. Although previous studies have found that inulin intervention does not significantly affect the behavioral phenotypes and molecular indices in healthy mice (Li et al., 2025; Liu et al., 2020), future studies including an inulin-only treatment group are essential to more precisely investigate the corrective or restorative effect of inulin under pathological conditions. Third, clinical studies have found that schizophrenia patients exhibit significant sex differences in prevalence, symptomatology, and treatment response (Moniem and Kafetzopoulos, 2025). Similarly, rodent models of schizophrenia also show sexually dimorphic behavioral phenotypes, microbiota profiles, and neurobiological alterations (Leger and Neill, 2016; Shobeiri et al., 2022). Therefore, both sexes should be included in future studies investigating the molecular mechanisms underlying chronic ketamine-associated schizophrenia-like behaviors. Fourth, SCFAs have been reported to promote the synthesis and secretion of BDNF in the CNS by inhibiting histone deacetylases (HDACs), reducing inflammatory responses, or directly binding to G protein-coupled receptors (GPCRs) (Suda and Matsuda, 2022; Ju et al., 2023). However, the exact mechanism by which SCFAs modulate the expression of BDNF following chronic ketamine exposure remains to be elucidated. Further studies using cellular methods or gene editing techniques are needed to clarify this underlying mechanism. Fifth, we did not conduct the SCFA intervention experiment, which may impact our investigation of the effects of SCFAs on chronic ketamine-induced schizophrenia-like behaviors. Previous studies have found that SCFA intervention alleviates constipation-induced depression and anxiety-like behaviors in mice (Zou et al., 2024). Further studies, such as the supplementation of exogenous SCFAs, are essential to elucidate the role of SCFAs in chronic ketamine-induced anxiety-like behaviors, deficits in PPI, and deficits in spatial learning and memory.

## 5 Conclusion

In summary, our results provide the first molecular and behavioral evidence that chronic ketamine induces anxiety-like behaviors, deficits in PPI, and deficits in spatial learning and memory partly by inducing gut microbiota dysregulation, reducing the expression of SCFAs, elevating gut permeability, downregulating the BDNF-TrkB-ERK1/2-CREB signaling pathway, and causing neuronal damage along with decreased expression of Syn and PSD-95. Notably, inulin intervention attenuates chronic ketamine-induced anxiety-like behaviors, impairments in PPI, and impairments in spatial learning and memory by restoring gut microbiota dysbiosis, increasing the expression of SCFAs, improving gut permeability, and upregulating the BDNF-TrkB-ERK1/2-CREB signaling pathway to reduce neuronal damage and increase the expression of Syn and PSD-95. Our study provides a potential strategy for ameliorating chronic ketamine-induced anxiety-like behaviors and deficits in spatial learning and memory. Our findings offer additional evidence that inulin may be a viable candidate for the treatment of chronic ketamine-induced anxiety-like behaviors, impairments in spatial learning and memory, and behavioral deficits in schizophrenia associated with dysbiosis.

## Data Availability

The data presented in the study are deposited in the National Center for Biotechnology Information (NCBI) repository, accession number: PRJNA1437400, https://www.ncbi.nlm.nih.gov/sra/PRJNA1437400.
