# Supplementary material for: Inulin ameliorates chronic ketamine-induced anxiety-like behaviors and impairments in spatial learning and memory: involvement of gut microbiota, microbial metabolite short-chain fatty acids, and the BDNF-TrkB-ERK1/2-CREB pathway
Source: Front Microbiol. 2026 Apr 7;17:1765079. doi: 10.3389/fmicb.2026.1765079 (PMC13095750; doi:10.3389/fmicb.2026.1765079)
Supplement: Supplementary file 1 [file Data_Sheet_1.docx]

***Supplementary materials***

**1. Antibodies used in Immunohistochemistry**

The following primary antibodies were used: anti-NeuN (GeneTex, California, USA, GTX01767; 1:100), anti-BDNF (GeneTex, California, USA, GTX132621; 1:250), anti-phospho-Tyr705-TrkB (Biosis, Beijing, China, bs-5526R, 1:200), anti-phospho-ERK1/2 (CST, Boston, USA, 4370; 1:200), anti-phospho-CREB (CST, Boston, USA, 9198; 1:400), anti-ZO-1(Abcam, Cambridge, UK, ab276131; 1:100)，and anti-Occludin (CST, Boston, USA, 91131; 1:100). The following secondary antibody was used: Alexa Fluor 488-conjugated donkey anti-mouse (Thermo Scientific, Rockford, USA, A-21202; 1:1000), Alexa Flour 488-conjugated donkey anti-rabbit (Thermo Scientific, Rockford, USA, A-21206; 1:1000), and Alexa Fluor 555-conjugated donkey anti-rabbit (Thermo Scientific, Rockford, USA, A-31572; 1:1000).

**2. Antibodies used in Western blot analysis**

The following primary antibodies were used: anti-PSD-95 (CST, Boston, USA, 3450; 1:1000), anti-Syn (CST, Boston, USA, 36406; 1:1000), anti-BDNF (Abcam, Cambridge, UK, ab108319, 1:800), anti-TrkB (CST, Boston, USA,4603, 1:500), anti-phospho-Tyr705-TrkB (Abcam, Cambridge, UK, ab229908, 1:500), anti-ERK1/2 (CST, Boston, USA, 4695; 1:1000), anti-phospho-ERK1/2 (CST, Boston, USA, 4370; 1:2000), anti-CREB (CST, Boston, USA, 9197; 1:1000), anti-phospho-CREB (CST, Boston, USA, 9198; 1:1000), anti-ZO-1 (Abcam, Cambridge, UK, ab276131; 1:500), anti-Occludin (CST, Boston, USA, 91131; 1:500), and anti-GAPDH (CST, Boston, USA, 5174; 1:1000). The following HRP-conjugated secondary antibody was used: goat anti-rabbit IgG (H+L) (CST, Boston, USA, 7074; 1:3000).

**3. Primer used in Real-time quantitative RT-PCR (qRT-PCR)**

The specific qRT-PCR primer sequences were as follows:

| BDNF | forward 5′- TCATACTTCGGTCATGAAGG-3′ |
| --- | --- |
|  | reverse 5′- ACACCTGGGTAGGCCAAGTT-3′ |
| TrkB | forward 5′- CTGGGGCTTATGCCTGCTG-3′ |
|  | reverse 5′- AGGCTCAGTACACCAAATCCTA-3′ |
| GAPDH | forward 5′-ACCACAGTCCATGCCATCAC-3′ |
|  | reverse 5′-ACCTTGCCCACAGCCTTG-3′ |

**4. Supplementary Figures**


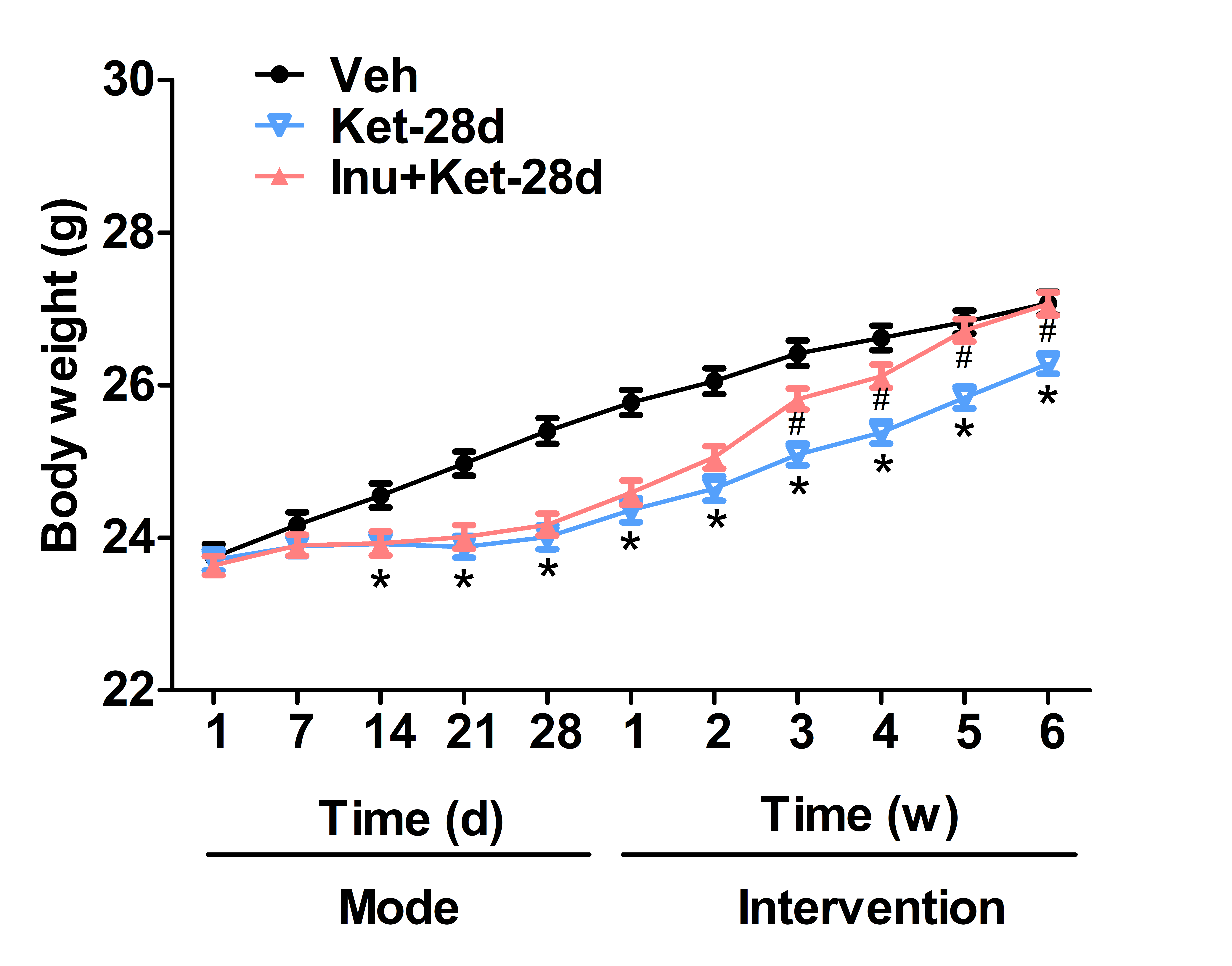


**Supplementary Figure 1.** Effects of chronic ketamine exposure and inulin intervention on the body weight. The body weight of mice was significantly decreased in mice injected with ketamine for 28 days, and this decrease was ameliorated by inulin treatment (n = 11). Data are expressed as mean ± SEM; **p* <0.05 vs the Veh group, ^#^*p* < 0.05 vs the Ket group. Veh, vehicle; Ket, ketamine; Inu, inulin; d, day; w, week.


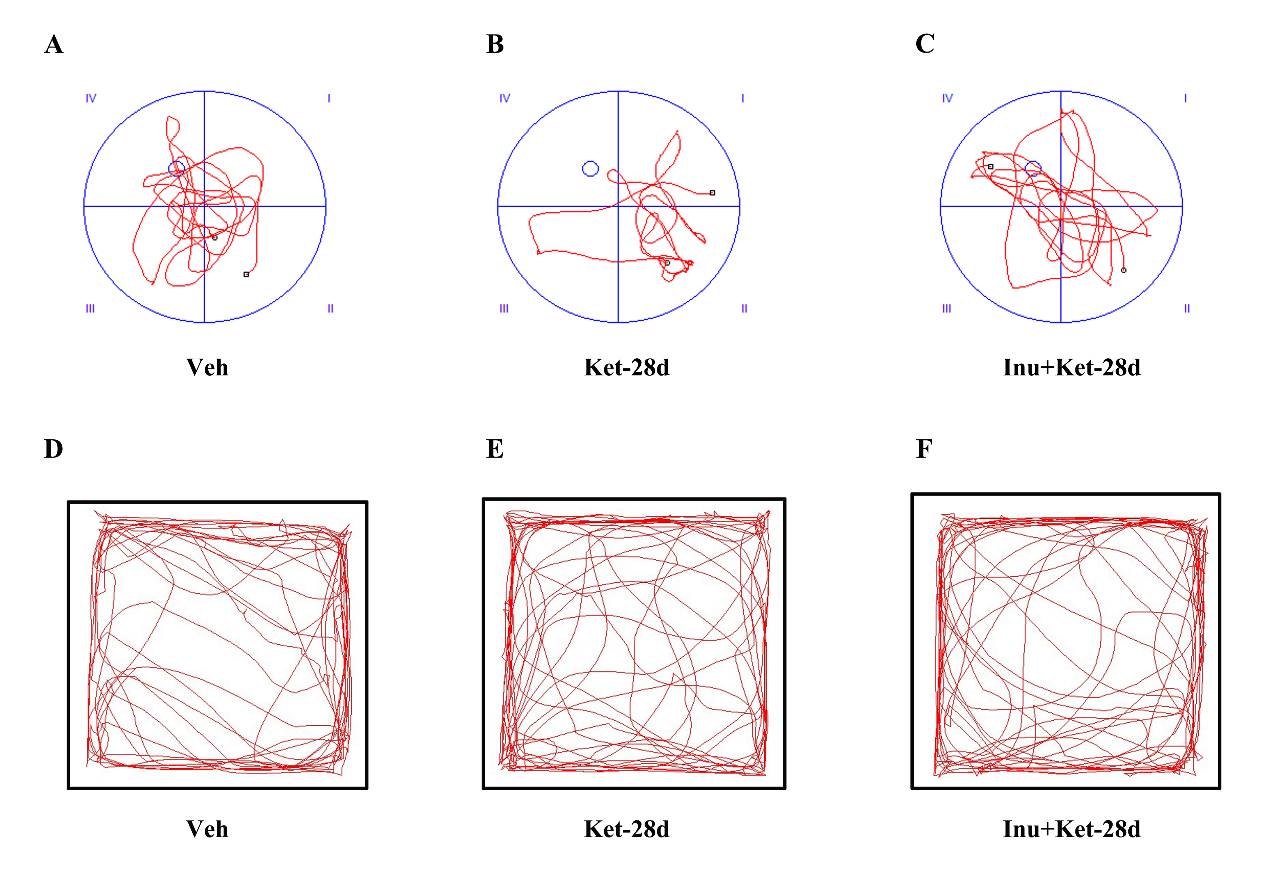
 **Supplementary Figure 2.** Representative trajectory graphs for behavioral test. (**A-C**) Mouse trajectory in the probe trial in Morris water maze (n = 11). (**D-E**) Mouse trajectory in open filed test (n = 11). Veh, vehicle; Ket, ketamine; Inu, inulin.


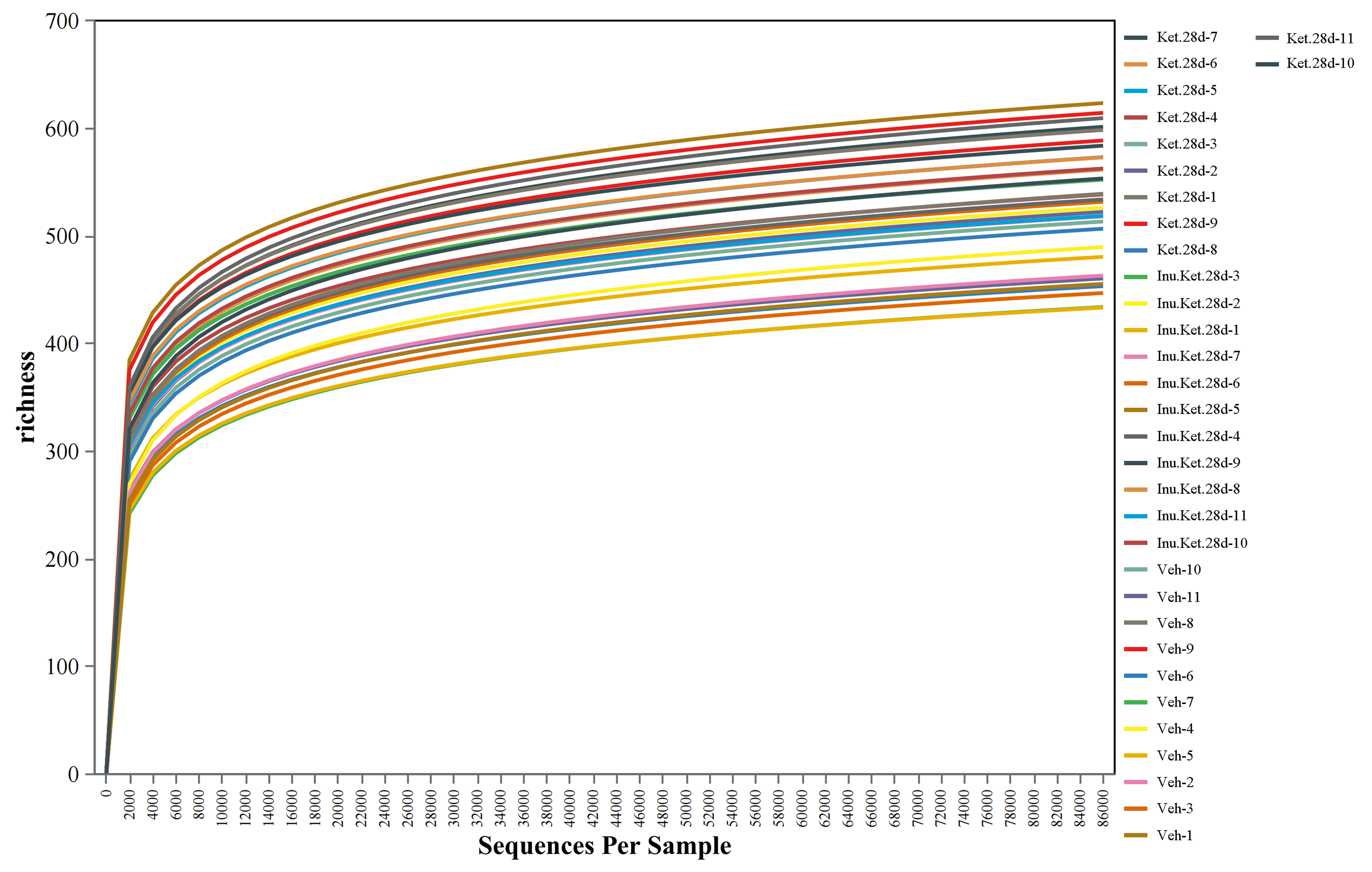


**Supplementary Figure 3.** The rarefaction curve analyses of sequencing depth adequacy of gut microbiota. The majority rarefaction curves approached the saturation plateau, suggesting that the sequencing depth of gut microbiota was adequate (n = 11). Veh, vehicle; Ket, ketamine; Inu, inulin.
